# Very Short Single-Acceptor Hydrogen Bonding in Liquid Chlorosulfonic Acid Enables Long Chain Formation

**DOI:** 10.1021/acs.jpclett.6c01833

**Published:** 2026-07-22

**Authors:** Miroslava Novoveska, Thomas F. Headen, Anna Herlihy, Camilla Di Mino, Neal T. Skipper, Daniel W. N. Wilson, Adam J. Clancy

**Affiliations:** † Department of Chemistry, 4919 University College London, 20 Gordon Street, London, WC1H 0AJ, U.K.; ‡ ISIS Neutron and Muon Source, Science and Technology Facilities Council, Rutherford Appleton Laboratory, Didcot, OX11 0QX, U.K.; § 120796 Diamond Light Source, Diamond House, Harwell Science and Innovation Campus, Fermi Ave, Didcot OX11 0DE, U.K.; ∥ Physical and Theoretical Chemistry Laboratory, Department of Chemistry, 6396 University of Oxford, South Parks Road, Oxford OX1 3QZ, U.K.; ⊥ Department of Physics and Astronomy, 4919 University College London, London, WC1E 6BT, U.K.

## Abstract

Chlorosulfonic acid is a superacid widely used as a sulfonating and chlorosulfonating agent. Here, we have used total neutron and X-ray scattering to understand its intra- and intermolecular structure, and interactions in the liquid phase. The behavior is dominated by very short hydrogen bonds between the hydroxyl groups and the sulfonyl oxygens (H···O^oxo^ 1.43 Å and 1.66 Å), comparable in length to intramolecular hydrogen bonds, with the resulting coordination number of 0.96 of sulfonyl oxygen around hydrogen below 2 Å. The hydrogen bonding leads to formation of branched head-to-tail chains and the intramolecular structure distorts to give three near-equal S–O bond lengths, consistent with the superacid’s low viscosity and high acidity.

## Graphical abstract

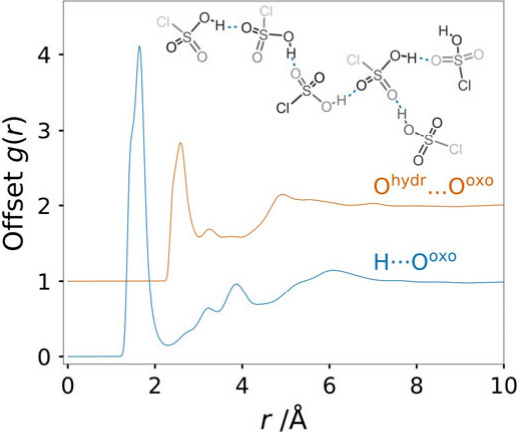

Chlorosulfonic acid (CSA, HSO_3_Cl) is a superacid structurally similar to sulfuric acid, with one hydroxyl replaced by an electron withdrawing chlorine, leading to higher Hammett acidity function (−13.80)^1^ of the remaining OH than in sulfuric acid. While its low p*K*_A_ (∼−5.9)^2^ means it is a very strong acid, its weak S–Cl bond makes it a common reagent in organic chemistry, where it is used as a sulfonating and chlorosulfonating agent for aromatics, alcohols, and amines.^3^ Similar to other protic solvents, CSA has a high dielectric constant (60 ± 10)^4^ with a moderate dipole moment (2.8 D).^5^ Thanks to these properties, CSA is widely used in commercial synthesis of pharmaceuticals,^6^ pesticides,^7^ artificial sweeteners (saccharin),^8^ surfactants,^8^ dyes,^8^ polymers,^3^ and ion-exchange resins,^6^ while it is also employed as a solvent, notably for otherwise-insoluble nanomaterials such as carbon nanotubes and graphene.^9–12^

Intermolecular interactions dictate the structure of liquids, which in turn controls their properties, behavior and reactivity. However, liquid structures cannot always be inferred from gas or solid phase studies^13–16^ and are often challenging to probe experimentally. As liquids are disordered systems with dynamically changing local structure, any diffraction measurements will yield a spatial and temporal average of all configurations and interactions in the system. Total neutron scattering (TNS) and total X-ray scattering (TXS) are powerful techniques for structure determination of disordered systems. TNS is sensitive to the position of hydrogen atoms, and as neutron scattering is isotopically dependent, it allows several scattering patterns to be acquired for the same chemical system through isotopic substitution, which provides information on site-specific local environments, inaccessible with other techniques. Furthermore, the existence of vanadium as a near-ideal incoherent scattering control allows simple and accurate normalization of scattering patterns onto an absolute scale, allowing numerical information such as coordination numbers to be extracted. When compared to TNS, TXS provides a highly distinct contrast scattering pattern, with emphasis on heavier atoms due to scattering from elements scaling with their atomic number (Z). Together, TNS and TXS therefore provide complementary data sets that allow the investigation of structural motifs containing both hydrogen and heavier atoms, separately.^17^

The intermolecular structures of acids are dominated by hydrogen bonds (HBs); X–H···Y interactions between electron-rich donor (X) and acceptor (Y) atoms/sites, characterized by their partially covalent character with a significant electrostatic contribution, lengthening of the X-H bond, and proclivity toward a 180° angle.^18^ The length of a hydrogen bond depends strongly on the X and Y chemistries, with strong hydrogen bonds (X/Y = N, O) typically having X···Y distances of 2.5–3.2 Å (H···Y, 1.5–2.2 Å). Acids form particularly short HBs, for example, sulfuric acid forms 2.7 Å long HBs (O···H, 1.7 Å).^19^ Chemicals with X and Y sites on the same molecule can form intramolecular HBs with notably shorter distances (e.g., picolinic acid N-oxide, 2.43 Å),^20^ with the most extreme example being the bifluoride anion at 2.30 Å.^21,22^

Unlike aqueous acids, which readily dissociate into hydronium and conjugate base, pure acids mostly exist as neutral molecules, with structures driven by HBs, for example, glacial acetic acid forms short chains of alternating perpendicular molecules stabilized by O–H···O^carbonyl^ HBs (H···Y, 1.67 Å; X···Y, 2.66 Å).^23^ Shorter HBs are seen in liquid fluorosulfonic acid,^24^ where two H···Y distances of 1.48 Å and 1.74 Å were measured by TNS, corresponding to X···Y 2.46 Å and 2.72 Å assuming a linear structure. In the solid state, fluorosulfonic acid (and the related triflic acid) adopts a linear chain motif of [−O–H···O=S−]_n_ with the sulfonyl oxo oxygen acting as the acceptor (X···Y, 2.64 Å).^25^

Despite its utility, CSA remains poorly understood, with previous works limited to determining the intramolecular structure by infrared and microwave spectroscopy in the gas phase, and computational studies.^5,26^ Since CSA is most commonly used as a liquid, it is important to understand the structure and bonding in this state, with particular questions remaining regarding its intramolecular structure in the liquid state, the nature of any intermolecular hydrogen bonding, and any long-range structuring of the molecules. Here, we have used total neutron and X-ray scattering to resolve the liquid structure of chlorosulfonic acid to understand its intermolecular interactions and rationalize its macroscopic properties.

Scattering data sets were obtained for room temperature (298 K) CSA using both total neutron scattering (three isotopologues: HSO_3_Cl, 50:50 HSO_3_Cl/DSO_3_Cl, and DSO_3_Cl) and total X-ray scattering. This approach provides contrast of both hydrogen and high-Z elements, allowing us to disentangle the intra- and intermolecular structures which occur on similar length scales (∼1.2–3.5 Å). Total neutron scattering experiments were performed using the Near and InterMediate Range Order Diffractometer (NIMROD)^27^ instrument at the ISIS Neutron and Muon Source (Didcot, UK), which is designed for studying disordered protium-rich samples. Total X-ray scattering was measured on XPDF (I15-1) at the Diamond Light Source (Didcot, UK), providing complementary data thanks to its compatible *Q*-range and resolution (full details and neutron and X-ray scattering theory are provided in Supporting Information (SI) S.1). HSO_3_Cl was obtained commercially while DSO_3_Cl was synthetised from D_2_SO_4_ and PCl_5_; the full procedure is described in SI S.2. The liquids were sealed into quartz ampules (6 mm ID, 8 mm OD) for neutron measurements, and into a borosilicate capillary (2 mm OD) for the X-ray measurement.

The atomistic structure of the liquid was obtained from *Dissolve* ^28^ by fitting all four measured contrasts simultaneously with data-driven Monte Carlo/molecular dynamics simulations with iteratively refined intermolecular potentials via the Empirical Potential Structure Refinement (EPSR) routine.^29^ Details of this analysis are provided in SI S.1. The measured and simulated total structure factors, *F*(*Q*), and total radial distribution functions, *G*(*r*), are plotted in Figure 1 and show excellent agreement between model and experimental data across both high *Q*/low *r* (*Q* ≳2 Å^–1^, *r* ≲3.5 Å, intramolecular and HB) and intermediate *Q* and *r* (intermolecular) ranges. The deviation at low *r* (≲1.2 Å) in the X-ray total radial distribution function is attributed to Fourier transform artifacts, which are more prevalent in the X-ray data due to lower *Q*_max_.

**Figure 1:**
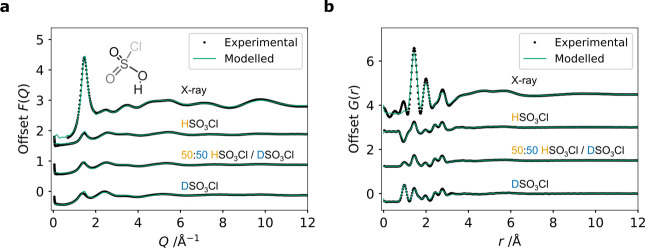
Total structure factors, *F*(*Q*), and total pairdistribution functions, *G*(*r*), for three total neutron scattering contrasts and one total X-ray scattering contrast of chlorosulfonic acid. The neutron structure factors are in barn atom^–1^ Sr^–1^ while the X-ray structure factor is unitless (normalized by the square of the average of all scattering contributions, SI S.1) N.B., *Q* range 0–12 Å^–1^ presented for clarity of dominant low-*Q* features. Full *F*(*Q*)­s and *G*(*r*)­s including modeling residuals and errors are available in Figure S3.

The first peak in *G*(*r*) is assigned to the O–H bond, as illustrated by its negative value in the hydrogenated HSO_3_Cl due to the negative scattering length of hydrogen (b_H_ = −3.74 fm; b_D_ = 6.67 fm). The correctly modeled intensities of this peak in the neutron contrasts indicate that CSA is almost fully undissociated, as neutron scattering is quantitative and hence dissociation would reduce the O–H peak area. An estimate of the amount of autoprotolysis based on specific electrical conductivity^4^ suggests the presence of one dissociated pair per ∼10^4^–10^5^ undissociated molecules, which is below the detection limit of the instruments in this study. Full peak assignments of the data and partial pair distribution functions are provided in the SI Figures S4 and S5. The intramolecular bond lengths and bond angles of liquid CSA extracted from our analysis (Figure 2a) differ significantly from the gas-phase structure.^5^ Here, the liquid CSA shows near-equivalent S–O bond lengths to both hydroxyl (O^hydr^) and sulfonyl (O^oxo^) oxygens—1.454 Å and 1.457 Å, respectively—and equal O–S–O angles (∼113°). This oxygen conformity is a marked departure from the known gas-phase structure which exhibits longer S–O^hydr^ (1.594 Å) and shorter S–O^oxo^ (1.438 Å) bonds, and a wider intersulfonyl angle (124°). The trend toward S–O equivalence implies a structure more akin to a delocalized deprotonated sulfonate, although the hydrogen remains covalently attached to a specific oxygen (O^hydr^–H = 0.977 Å). A full comparison of intramolecular parameters between the gas phase structure, the DFT-optimized structure, and the neutron scattering derived structure is provided in SI Table S4.

**Figure 2:**
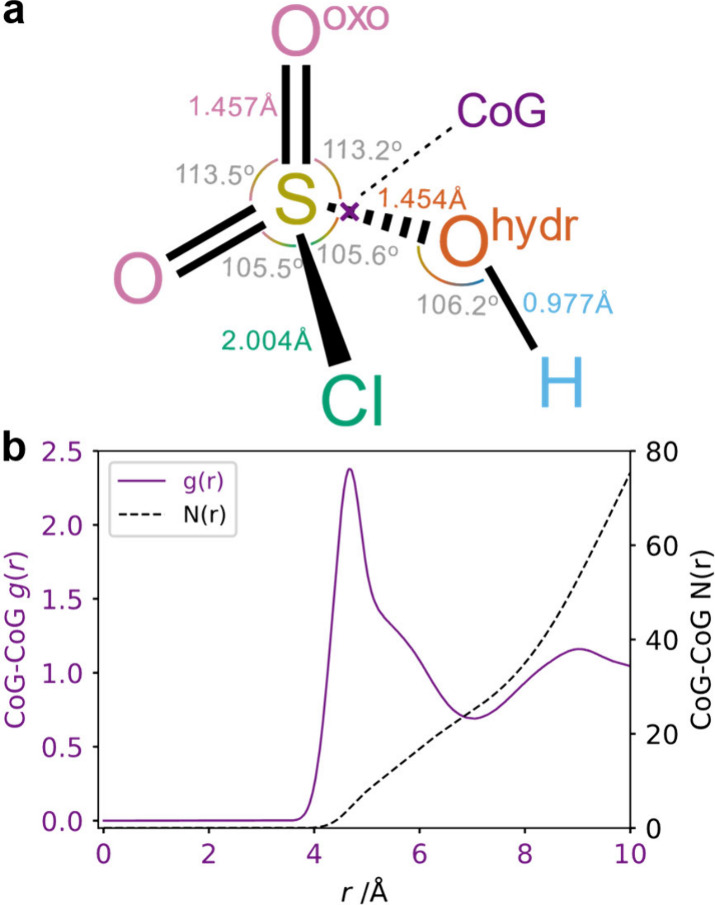
(a) Intramolecular bond lengths and bond angles of liquid CSA derived from neutron scattering-constrained modeling. Please note the bond length similarity between S–O^hydr^ and S–O^oxo^ and the strong intermolecular interactions within the system that may be due to a change in the bond order; their depiction as “single” and “double” has been kept from the gas phase structure of the acid here. (b) Radial distribution function, *g*(*r*), and running coordination number, *N*(*r*), for CSA center-of-geometry to center-of-geometry. Related coordination numbers are provided in SI Table S10.

The CSA liquid structure is highly ordered, with a sharp first solvation shell peak seen in the center-of-geometry (CoG) radial distribution function (Figure 2b, *g*(*r*)_max_/*g*(*r*)_min_ = 3.45), with a maximum intensity at 4.67 Å and a coordination number of 12.7 below the first minimum (7.05 Å). The structure is dominated by intermolecular HBs with the H atom almost exclusively binding to one of the two sulfonyl oxo oxygen atoms, O^oxo^ (H···O^oxo^ coordination number 0.96 ± 0.02, <2.0 Å), at two distinct H···O^oxo^ distances, 1.43 Å and 1.66 Å (high intensity features in Figure 3a, O^hydr^···O^oxo^ 2.41 Å and 2.59 Å) at 1 to ∼5.5 ratio. HB lengths and ratio were obtained by Gaussian fitting to 4*πρr* ^2^ *g*(*r*) and the presence of two peaks can be directly inferred from experimental data without reference to simulation (Figure S6). A full discussion on the assignment of the two HB environments is provided in SI S.5. Both motifs are notably longer than the H–O^hydr^ intramolecular bond (0.98 Å), highlighting that the H atom is primarily associated with only one O atom. Both of the HBs present in CSA are among the shortest measured, being significantly shorter than sulfuric acid (H···O^oxo^ ∼1.7 Å, O^hydr^···O^oxo^ 2.7 Å).^19^ The shorter of the two CSA HBs is shorter than many intramolecular HBs and only 0.29 Å longer than bifluoride (1.14 Å, F···F 2.3 Å),^21,22^ the shortest known HB interaction. Very short and strong HBs are consistent with the very high acidity of CSA.

**Figure 3:**
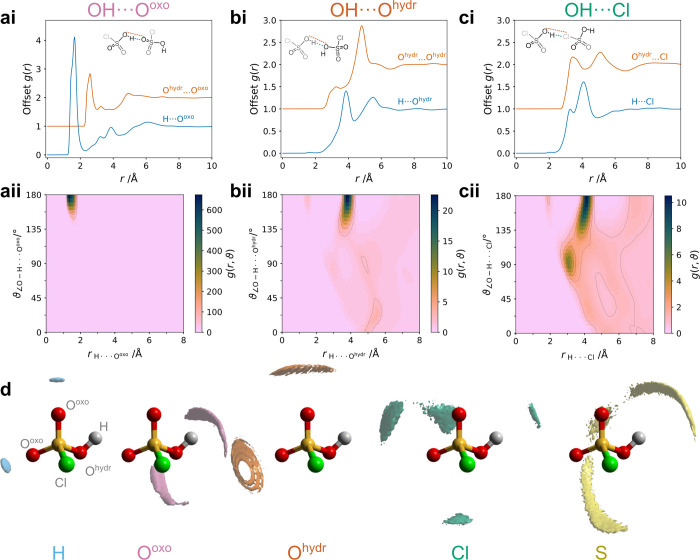
(a–c) Partial radial distribution functions for H···Y and O^hydr^···X distances (i) and angle-distance maps for H···Y distances and ∠O–H···Y angles, each for Y being (a) O^oxo^, (b) O^hydr^, (c) Cl. High intensity features correspond to correlations due to O^hydr^–H···O^oxo^ hydrogen bonding. O^hydr^-H···O^hydr^ and O^hydr^-H···Cl hydrogen bonds only contribute very small features close to H···Y 2 Å in (b) and (c). (d) Spatial distribution functions of individual CSA atoms around an averaged CSA molecule. Top 10% most likely atom positions, with distributions centered on sulfur. N.B., the position of the H atom in the central averaged CSA molecule is not fixed, the H atom can rotate around the S–O^hydr^ bond and the SDFs contain contributions from all of its possible positions. Cutoffs: S–H: 3.4 Å, S–O^oxo^: 3.8 Å, S–O^hydr^: 3.8 Å, S–Cl: 4.0 Å, S–S: 4.4 Å. Peak assignment to bonding motifs in angle-distance maps are provided in SI S.6.

The O–H···O^oxo^ angle is near linear, centered around 180° (Figure 3aii), and it is colinear with the acceptor-oxygen’s S=O bond (Figure 4a). The dominance of the O–H···O^oxo^ interaction can be seen in the spatial distribution function (SDF), with strong localization of H around the O^oxo^ atoms. The O^hydr^ are also subsequently localized in a toroidal region surrounding hydrogen due to the O^hydr^–H intramolecular bond (Figure 3d).

**Figure 4:**
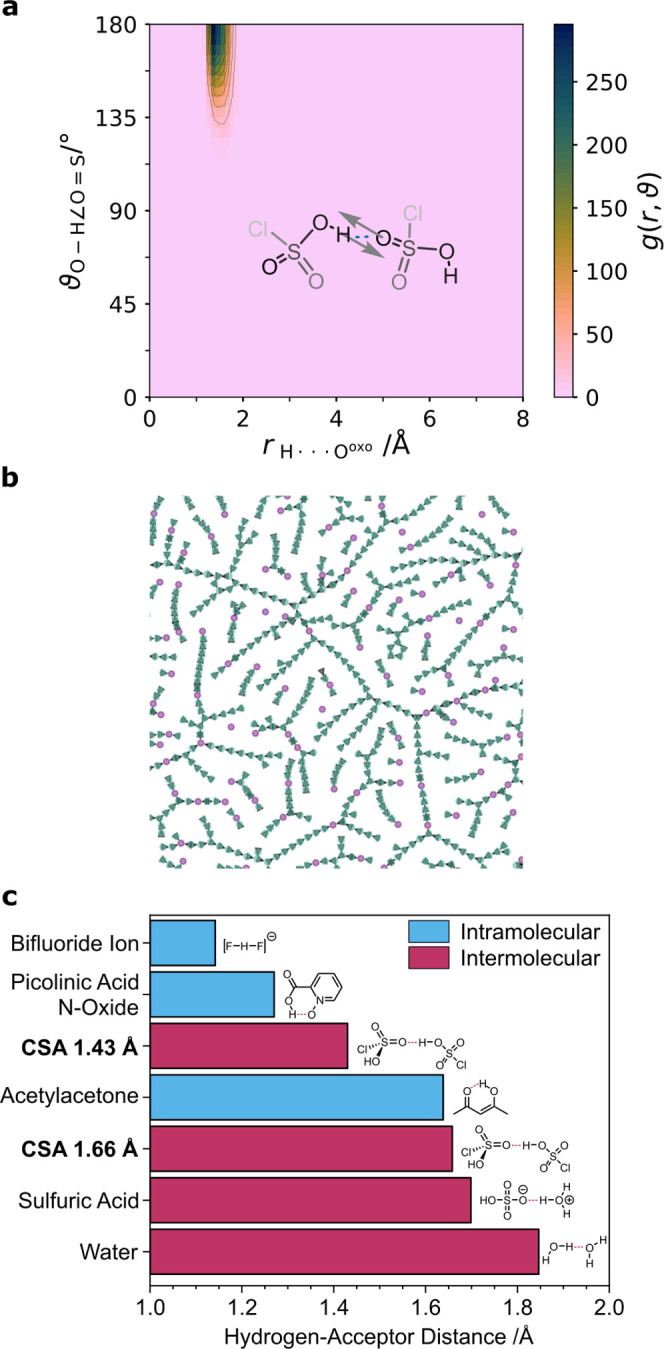
(a) Angle-distance map of the angle between O–H and S=O bonds (intermolecular) as a function of H···O­(=S) distance. (b) Representative hydrogen bonded chlorosulfonic acid chains with O^hydr^···O^oxo^ < 3.0 Å and O–H···O^oxo^ > 150°, extracted from one frame of the experimentally constrained model with 3663 CSA molecules. These cutoff values were selected from minima of the *g*(*r*) and ARDF to maintain strict criteria for confirming chain formation, and molecules interacting with weaker or less directional HBs will not be captured. No O–H···O^hydr^ or O–H···Cl interactions were present in this frame. Molecules donating HB are represented by green arrows, molecules not donating HB by pink circles. Note that the 3D spatial orientation of the chains has not been preserved, only the chain connectivity. (c) Common hydrogen bonding distances between two donor atoms (i.e., O···O for all systems but [F–H–F]^−^). Acetylacetone and picolinic acid N-oxide are molecules with intramolecular hydrogen bonds. [F–H–F] ^–^ is the strongest known hydrogen bonding system.^19–21,31,32^

While HBs in CSA also form at both O^hydr^ and Cl hydrogen-acceptor sites (O^hydr^···O^hydr^, 2.55 Å, H···O^hydr^ coordination number 0.0023 ± 0.0009, <2.0 Å; O^hydr^···Cl, 2.85 Å, H···Cl coordination number 0.0048 ± 0.0011, <2.35 Å) with ideal 180° HB angles, the probability of these interactions is approximately two to three orders of magnitude lower than HB formation with an O^oxo^ acceptor (Figure 3bii, cii, SI Figure S15). The loci of the Cl and O^hydr^ atoms are instead primarily dictated by the H···O^oxo^ HBs, and all other features in the angle-distance maps and SDFs have been found to be linked to the consequent constraint of the molecular orientation. Detailed assignments are provided in SI S.6. The coordination number cut-offs correspond to minima in angle-distance distributions.

Interactions of one H atom with two acceptor sites at once (bifurcated HBs) are transient: there is a clear preference for the H atom to interact with only one acceptor site at a time (three orders of magnitude difference for O^hydr^···O^oxo^ < 3.5 Å and O^hydr^–H···O^oxo^ > 150°). As CSA molecules are inherently limited to one H-donating site, the single-acceptor nature of the hydrogen leads to the formation of chains, akin to the linear chains seen for solid fluorosulfonic and triflic acids.^25^ While disfavored, a notable fraction of the CSA molecules partakes in HB via multiple acceptor sites – primarily with two O^oxo^ hydrogen acceptors (∼0.15 of CSA molecules accept two H···O^oxo^ bonds, Figure 4b). These double-H acceptor molecules can exist either in the middle of chains (donating 0 HBs) with chain direction reversed either side, or form tribonded junctions (donating 1 HB), leading to the formation of sparsely branched chains, which mostly interact via van der Waals interactions, leading to the much lower (by an order of magnitude) viscosity of liquid CSA compared to sulfuric acid.^4,30^ As sulfuric acid provides two H-donors and two H-acceptors, it can form more interconnected 3D hydrogen bonded networks, with slower flow than the more linear CSA chains which can slip past each other under shear. More details on CSA chain formation are provided in SI S.7.

To summarize, we have determined the liquid structure of chlorosulfonic acid, a very strong acid useful in a range of applications. We have found that hydrogen bonding is by far the dominant interaction in the system, which occurs almost exclusively with the sulfonyl oxo oxygen (S=O) as the hydrogen-acceptor. The formed hydrogen bonds, 1.43 Å and 1.66 Å, are among the shortest ever measured, comparable to intramolecular hydrogen bonds (Figure 4c) and only slightly longer than the shortest hydrogen bonds known to exist in bifluoride. The hydrogen bonds are highly directional and constrain the O–H···O=S angle to near-linear, and lead to the formation of branched chains of molecules in the liquid. The interesting chemical and physical properties of this acid are linked to the nature of these hydrogen bonds, from its high boiling point to the low viscosity. The CSA molecules themselves distort due to formation of these hydrogen bonding chains, with equivalence of the S–O^hydr^ and S=O^oxo^ bond lengths in contrast to the gas phase where the S–O^hydr^ is 0.14 Å longer. As our study has also shown how strongly the intra- and intermolecular interactions are linked to the liquid environment, the chosen solvent in organic reactions involving chlorosulfonic acid, or the solute when chlorosulfonic acid is used as a solvent, will have a strong impact on the properties and reactivity of the acid. This knowledge will help to find new and understand the current uses of this superacid, while providing experimental data sets of liquid CSA against which to benchmark ongoing and future computational studies.^33^

## Supplementary Material



## Notes

Neutron scattering theory including per-atom scattering weightings, experimental procedure, full-range *F*(*Q*) and *G*(*r*), supporting (A)­RDFs data, and expanded discussion on the hydrogen bonding double peak. (PDF)
