# Supplementary material for: Very Short Single-Acceptor Hydrogen Bonding in Liquid Chlorosulfonic Acid Enables Long Chain Formation
Source: J Phys Chem Lett. 2026 Jul 22;17(31):9025–30. doi: 10.1021/acs.jpclett.6c01833 (PMC13455093; doi:10.1021/acs.jpclett.6c01833)
Supplement: Supplementary file 1 [file jz6c01833_si_001.pdf]

Supporting Information for Very Short Single-Acceptor Hydrogen Bonding in Liquid Chlorosulfonic Acid Enables Long Chain Formation

Miroslava Novoveska[a], Thomas F. Headen[b], Anna Herlihy[c], Camilla Di Mino[d], Neal T. Skipper[e]*, Daniel W. N. Wilson[a]*, Adam J. Clancy[a]*

[a] Department of Chemistry, University College London, 20 Gordon Street, London, WC1H 0AJ, UK

[b] ISIS Neutron and Muon Source, Science and Technology Facilities Council, Rutherford Appleton Laboratory, Didcot, OX11 0QX, UK

[c] Diamond Light Source, Diamond House, Harwell Science and Innovation Campus, Fermi Ave, Didcot OX11 0DE, UK

[d] Department of Chemistry, University of Oxford, South Parks Road, Oxford OX1 3QZ, UK

[e] Department of Physics and Astronomy, University College London, London, WC1E 6BT, UK

Table of Contents

S.1 Neutron and X-ray scattering theory 2

S.2 Experimental procedure 11

S.3 Total structure factors ($F(Q)$) and total radial distribution functions ($G(r)$) 13

S.4 Radial distribution functions (RDFs) and coordination numbers 15

S.5 Hydrogen bonding double peak 16

S.6 Angular radial distribution functions (ARDFs) 20

S.7 Chain formation and HB coordination numbers 22

References 24

S.1 Neutron and X-ray scattering theory

Total neutron and X-ray scattering take advantage of coherent elastic scattering from the sample, with no energy loss and with *Q*-dependence reflecting the structural correlations in the material, where *Q* is the magnitude of the momentum transfer between the incoming and scattered neutron/photon. Preserved *Q*-dependence It is also assumed that the measured signal purely corresponds to single-scattering events, rather than multiple scattering. Deviation from these assumptions, i.e. contributions from incoherent and inelastic scattering (neutrons), fluorescence (X-rays), and multiple scattering (both) are corrected for.[1] Basic scattering theory is discussed in *Sivia et al*.[2]

The neutron coherent scattering cross-sections differ significantly and irregularly for different isotopes. There is no clear general trend across the periodic table (apart from a gradual increase for heavier elements), with a large difference between light hydrogen, ^{1}H, (b_H = -3.739 fm) and deuterium, ^{2}D, (b_D = 6.671 fm) utilized in isotopic substitution. Isotopic substitution allows acquisition of multiple different scattering patterns from chemically equivalent samples, highlighting different contributions to the scattering pattern. Neutron scattering is particularly useful for hydrogen-rich samples.

Scattering intensity of X-rays is governed by the form factor which has a strong, linear dependence on the number of electrons of an atom, and hence the atomic number *Z*. The form factor of H is therefore negligible, with the X-rays scattering pattern more sensitive to heavier elements (in our case S, Cl).

NIMROD, the Near and InterMediate Range Order Diffractometer at ISIS Neutron and Muon Source, has been specifically designed for measurements of hydrogen-rich samples. It is able to measure signal in the *Q*-range 0.02-50 Å^{-1}, bringing <0.1 Å resolution to *r* and a maximum measurable structure size to ~300 Å.[3,4]

I15-1, the X-ray Pair Distribution Function (XPDF) beamline at Diamond Light Source, mainly operates at 76.6 keV with a broad measurement *Q*-range 0.65-20 Å^{-1}, compatible with the neutron scattering data.[5,6]

Total neutron scattering data were processed in *GudrunN*, equivalently, the differential X-ray scattering cross-section was processed in *GudrunX*.[1] The resulting total structure factors, *F(Q)*, are reciprocal space functions directly related to structural correlations. The neutron structure factors differ for different isotopic compositions as neutron scattering length, $\bar{b}$, is isotope-sensitive.

$$F(Q) = \sum_{i,j\geq i}\left(2-\delta_{ij}\right)c_i c_j \bar{b}_i \bar{b}_j \left(S_{ij}(Q)-1\right), \quad \text{(S.1)}$$

where c_i, c_j are the atomic fractions of atom types *i,j*; $\bar{b}_i$, $\bar{b}_j$ are the neutron scattering lengths of appropriate isotopes of atom types *i,j*; *Q* is magnitude of the scattering vector, and $S_{ij}(Q)$ is the partial structure factor of atom types *i,j* defined by

$$S_{ij}(Q) - 1 = \frac{4\pi\rho_0}{Q}\int_0^\infty r\left[g_{ij}(r) - 1\right]\sin(Qr)\,dr, \qquad \text{(S.2)}$$

where ρ_0 is the atomic number density of the system, *Q* is the momentum transfer vector defined as the change in the momentum of the scattered particles, *r* is the interatomic distance; and g_{ij} is the pair distribution function of atom types *i,j*. $S_{ij}(Q)$ and $g_{ij}(r)$ are related by Fourier transform and hence are each other's equivalent in reciprocal and real space, respectively.

Table S1. Neutron scattering lengths for H, D, O, S, and Cl.[7]

	H	D	O	S	Cl
b_i/fm	-3.74	6.671	5.803	2.847	9.577

X-ray total structure factors obey equation S.3

$$F(Q) = \sum_{i,j\geq i}\frac{(2-\delta_{ij})c_i c_j f_i(Q) f_j(Q)\left(S_{ij}(Q)-1\right)}{\langle\sum_{i=1}^{n} c_i f_i(Q)\rangle^2}, \qquad \text{(S.3)}$$

where the X-ray scattering form factors are Fourier transforms of the atomic electron density, which can be represented by

$$f_i(Q) = \sum_{i=1}^{5}\alpha_i e^{-\beta_i\frac{Q}{4\pi}} + \gamma, \qquad \text{(S.4)}$$

and are evaluated at *Q* = 0, where they also equal to the number of electrons of each element.

Table S2. X-Ray scattering form factor parameters for O, S, and Cl used in Dissolve.[8]

	α_1	β_1	α_2	β_2	α_3	β_3	α_4	β_4	α_5	β_5	γ
O	2.9604	14.1823	2.5088	5.9369	0.6379	0.1127	0.7228	34.9585	1.1428	0.3902	0.027
S	6.3722	1.5143	5.1546	22.0925	1.4737	0.0613	1.6351	55.4452	1.2094	0.6469	0.1547
Cl	1.4461	0.0525	6.8706	1.1932	6.1518	18.3434	1.7503	46.3984	0.6342	0.4010	0.1468

$$g_{ij}(r) = 1 + \frac{1}{2\pi^2\rho_0}\int_0^\infty Q^2\left[S_{ij}(Q) - 1\right]\frac{\sin Qr}{Qr}dQ. \qquad \text{(S.5)}$$

$$G(r) = \frac{1}{2\pi^2\rho_0}\int_0^\infty Q^2F(Q)\frac{\sin Qr}{Qr}dQ = \sum\nolimits_{i,j\geq i}\left(2-\delta_{ij}\right)c_ic_j\bar{b}_i\bar{b}_j\left[g_{ij}(r) - 1\right] \qquad \text{(S.6)}$$

The weightings w_{ij} of $S_{ij}(Q) - 1$ in the measured $F(Q)$ are determined by atomic concentrations and scattering lengths as can be seen from equation S.1, and so can be expressed as

$$w_{ij} = \left(2-\delta_{ij}\right)c_ic_j\bar{b}_i\bar{b}_j. \qquad \text{(S.7)}$$

The weightings for all CSA contrasts are displayed in Table S3.

When the number of measured isotopic compositions is equal to (or larger than) the number of different atomic pairs ($N = n(n+1)/2$), $S_{ij}(Q)$ can be theoretically obtained from the measured $F(Q)$s by a series of N equations ($F(Q)$s) with N unknowns ($S_{ij}(Q)$) without any knowledge of the system apart from its isotopic composition, and subsequently converted via Fourier transform (FT) into pairwise site-site correlations in real space. However, in practice, this method is susceptible to introducing unphysical solutions (since the system is largely unconstrained and does not take into account physicochemical properties of the system such as density) and can introduce artefacts into the real-space data from the FT and from incompletely subtracted inelastic scattering in the $F(Q)$. Furthermore, in a more complex system, the requirement of $n(n+1)/2$ isotopic contrasts is often not possible to fulfil as the number of $S_{ij}(Q)$ contributions is large (in the case of CSA, 15 for a system with 5 distinct atomic sites) and the isotopic contrasts become too similar for small differences in composition.

Therefore, data-driven simulations are used for neutron scattering data analysis. In these simulations, atomic composition together with known molecular structures, system density, and seed potentials (input potentials matching physical properties of the system components) are used to predict each of the $S_{ij}(Q)$ contributions, which are subsequently refined using the measured $F(Q)$s in a routine called Empirical Potential Structure Refinement (EPSR). This approach offers a more reliable and detailed analysis route, validating the physicality of the real-space correlations and allowing a wider range of analysis. This routine and its implementation is described in detail in an article about the used software, *Dissolve*.[9,10]

The analysis routines of the simulation box include correlation functions such as radial distribution functions (S.3), angular radial distribution functions (ARDFs, S.6), and spatial density functions (SDFs), and coordination numbers. ARDFs are defined as

$$g(r,\theta) = \frac{\Delta n(r,\theta)}{\frac{2}{3}\pi((r+\Delta r)^3 - r^3)\rho \sin(\theta)\Delta\theta} \quad \text{(S.8)}$$

with $\Delta n(r,\theta)$ being the number of atoms/sites at distance *r* within Δr and angle θ within bin width $\Delta\theta$, and ρ as the atomic number density. The ARDFs represent the likelihood of finding an atom/site at distance *r* and ϑ from an atom/site at the origin. In order to evaluate the ARDFs, three sites within a system or a set of cartesian axes for each molecule of interest need to be defined (such as in Figure 4a in Main text). Similarly, the Spatial Density Functions (SDFs) are spherical harmonic representations of many-body correlation functions and provide a three-dimensional picture of the arrangement of objects around a central species as a function of their relative orientation.[1] Coordination numbers represent the number of atoms/sites that surround a given atom/site (placed at the origin) and are defined as:

$$N_{ij}(r_{\text{min}}, r_{\text{max}}) = 4\pi\rho_0 \int_{r_{\text{min}}}^{r_{\text{max}}} r^2 g_{ij}(r)dr. \quad \text{(S.9)}$$

Traditionally, the r_{max} of the integral in equation S.7 is the position of the first minimum of $g_{ij}(r)$ after r_{min}. Thus, the region between two consecutive minima is known as a coordination shell.

Table S3. Weighting factors (in barns, b, for neutrons, dimensionless for X-rays) of atomic pairs contributing to structure factors, F(Q), in CSA.

	HSO_3Cl	50:50 HSO_3Cl/DSO_3Cl	DSO_3Cl	X-ray
**S-S**	0.00198	0.00198	0.00198	0.06519
**S-O^{hydr}**	0.00403	0.00403	0.00403	0.03190
**S-O^{oxo}**	0.00806	0.00806	0.00806	0.06380
**S-Cl**	0.00665	0.00665	0.00665	0.06938
**S-H**	-0.00260	0.00102	0.00463	0.00399
**O^{hydr}-O^{hydr}**	0.00821	0.00821	0.00821	0.01561
**O^{hydr}-O^{oxo}**	0.01642	0.01642	0.01642	0.03122
**O^{hydr}-Cl**	0.01355	0.01355	0.01355	0.03395
**O^{hydr}-H**	-0.00529	0.00207	0.00944	0.00195
**O^{oxo}-O^{oxo}**	0.03285	0.03285	0.03285	0.06243
**O^{oxo}-Cl**	0.02711	0.02711	0.02711	0.06789
**O^{oxo}-H**	-0.01058	0.00415	0.01888	0.00390
**Cl-Cl**	0.02237	0.02237	0.02237	0.07384
**Cl-H**	-0.00873	0.00342	0.01558	0.00424
**H-H**	0.00341	0.00052	0.01085	0.00024

The modelled system consisted of chlorosulfonic acid molecules in a box (96.08 Å×96.08 Å×50.57 Å, α = β = 90° and γ = 120°) with atomic density 0.05436 atoms×$Å^{-3}$.[11] The OPLS-AA (Optimized Potentials for Liquid Simulations – All Atom) forcefield used for the initial 'seed' potentials was obtained from LigParGen[12–14] and the initial intramolecular structural parameters were initially obtained from DFT (geometry optimization in Orca 5.0 using B3LYP functional, DEF2-SVP basis set, Conductor-like Polarizable Continuum Model (ε = 60, refractive index = 1.433), and input coordinates from UFF relaxed single molecule structure in Avogadro2),[15–18] the heavier-atom bond lengths and bond angles were then adjusted according to the intramolecular part of X-ray and neutron scattering *G(r)*, while the O-H bond and H⋯X correlations only according to the neutron scattering data. (Table S4) The difference between the intramolecular structure in the liquid phase compared to gas phase/DFT is due to the presence of strong intermolecular interactions, which are not present in the gas phase and not sufficiently approximated by simulation as a dielectric medium in DFT. The near-equivalent S-O bond lengths are caused by the shortening of S-O^{hydr} from H-donation (1.594 Å → 1.457 Å) and S-O^{oxo} lengthening from H-acceptance (1.438 Å → 1.454 Å). The *G(r)*s show only a single sharp peak at ~1.45 Å in all four contrasts. (Main text Figure 1) There is a peak corresponding to intramolecular H⋯X distances in the *G(r)*s at ~3.20 Å which can only be caused by a restricted intramolecular torsion due to strong intermolecular interactions. We initially attempted to constrain the X-S-O-H dihedral angle but no constraint gave a significantly better overall fit. While the preferential orientations measured previously in the gas phase[19] reproduce this peak with EPSR off, the rest of the fit significantly worsens (including peak at ~2.8 Å also directly containing torsion correlations). Therefore, we have decided to remove any torsional constants so that the EPSR/MC/MD routine can freely adjust it to the experimental data as this approach should provide a more reliable result than the constraints. (Figure S1, Table S4, S5)

Furthermore, the analysis also allows to investigate the intermediate structure of the liquid to determine e.g., the extent of hydrogen bonding chains and (non)percolating networks, by defining ranges of distances and angles representing the bonding motifs of interest.

Table S4. Intramolecular parameters of chlorosulfonic acid. The table contains: Parameters corresponding to no solvation (i.e., gas phase) were taken from a microwave spectroscopy study,[19] parameters from a solvation by a dielectric medium were obtained from DFT run in Orca 5.0,[15–18] input molecular parameters to achieve reasonable initial fit were obtained by modification of the DFT parameters to match the intramolecular peaks in TXS and TNS, final intramolecular parameters that were obtained after running the EPSR routine and obtaining the fit in Main text, Figure 1. Note that the final intramolecular parameters listed in this table correspond to the maxima of Gaussian distributions of the parameters present in the simulation.

Parameters	Molecular solvation (TNS) – distribution max	Gas phase (microwave spectroscopy)		Computational (DFT, MP2 - CPCM)		Molecular solvation – input parameters	
		Value	Δ vs TNS	Value	Δ vs TNS	Value	Δ vs TNS
**Bond lengths /Å**							
$O^{(H)}$-H	**0.977**	0.972	-0.005	0.981	0.004	0.965	-0.012
S-$O^{(H)}$	**1.454**	1.594	+0.140	1.599	0.145	1.466	+0.012
S=$O^{(1)}$	**1.457**	1.438	-0.019	1.450	-0.007	1.455	-0.002
S=$O^{(2)}$	**1.457**	1.430	-0.027	1.446	-0.011	1.455	-0.002
S-Cl	**2.004**	2.039	+0.035	2.100	0.096	2.010	+0.006
**Bond angles /°**							
S-$O^{(H)}$-H	**106.2**	107.8	+1.6	111.3	+5.1	109.0	+2.8
$O^{(H)}$-S=$O^{(1)}$	**113.2**	109.3	-3.9	111.2	-2.0	106.7	-6.5
$O^{(H)}$-S=$O^{(2)}$	**113.2**	106.4	-6.8	105.9	-7.3	106.7	-6.5
$O^{(H)}$-S-Cl	**105.6**	100.4	-5.2	100.8	-4.8	100.0	-5.6
$O^{(1)}$=S=$O^{(2)}$	**113.5**	124.1	+10.6	122.6	+9.1	106.7	-6.8
$O^{(1)}$=S-Cl	**105.5**	106.8	+1.3	106.8	+1.3	100.0	-5.5
$O^{(2)}$=S-Cl	**105.5**	107.4	+1.9	107.4	+1.9	100.0	-5.5
**Dihedral angles /°**							
Cl-S-$O^{(H)}$-H	**~56, 105 (main)**	86.0	-19.0	76.1	-28.9	n/a	n/a

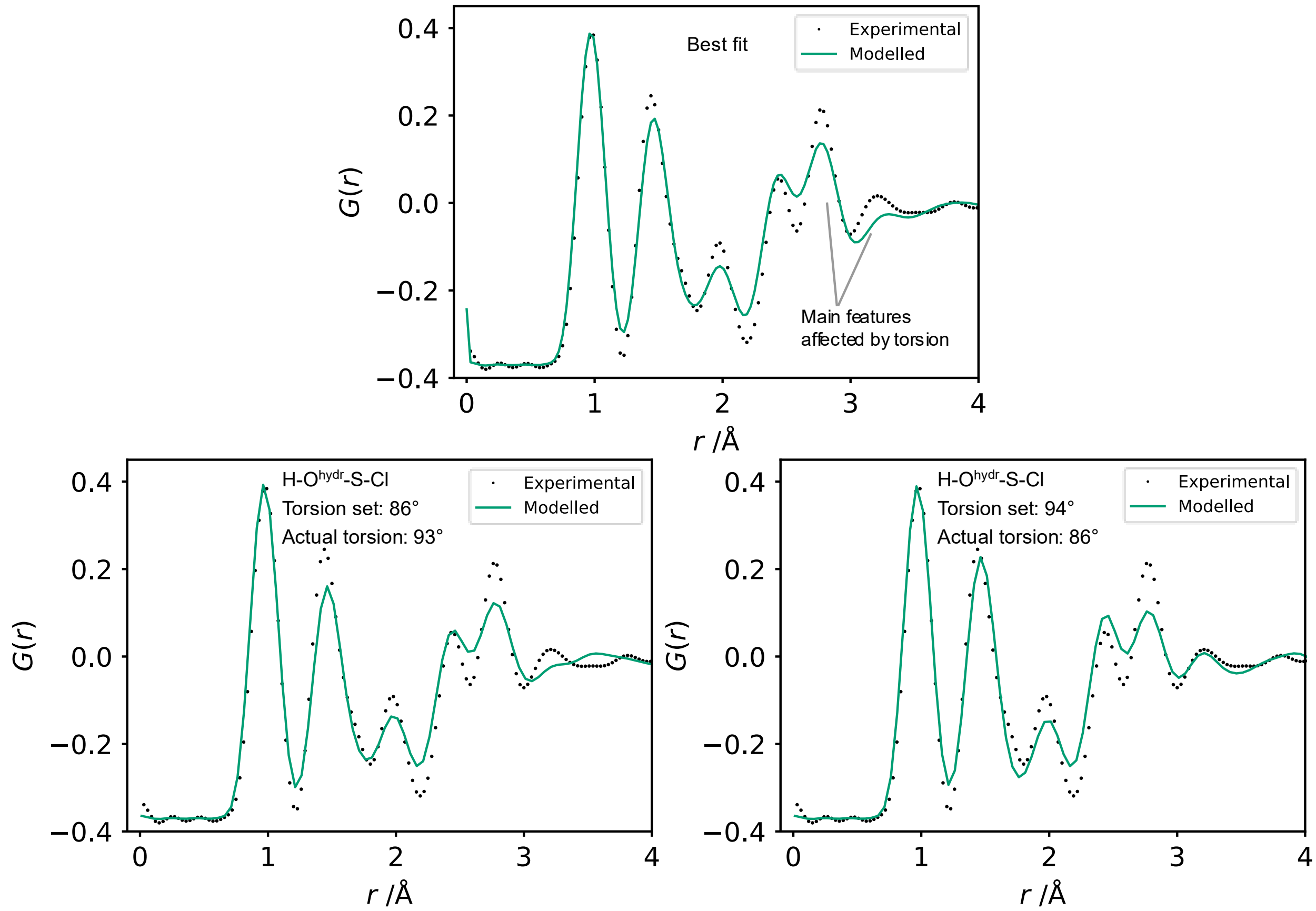

Figure S1. Best fit (with unconstrained torsion) versus fits with constrained H-O^{hydr}-S-Cl torsional angle demonstrated on DSO_3Cl contrast. Set torsion corresponds to the energy minimum for the H-O^{hydr}-S-Cl torsional angle set in the seed potential. Actual torsion is the average (and most probable) torsional angle sampled directly from the simulation box. Note that the fits with constrained torsion are both with EPSR off, while the best fit was obtained with EPSR on.

Table S5. Intermolecular potential parameters used in simulations.

Atom type	Charge / e	Potential	ε (potential well depth) / kJ/mol	σ (distance where potential is zero) / Å
**S**	1.4454	Lennard-Jones	1.1464	3.595
**O^{hydr}**	-0.6756		0.25104	3.118
**O^{oxo}**	-0.4949		0.25104	3.118
**Cl**	-0.3278		0.949768	3.5164
**H**	0.5478		0	0

Fractional contributions of atom types in all four scattering contrasts are provided in Tables S6-S9.

Table S6. Fractional neutron scattering contributions of individual atom-atom pairs in HSO_3Cl.

			Species	HSO_3Cl					Percentage Scattering Contribution
				S	O^{hydr}	O^{oxo}	Cl	H	
			Atom Fraction	0.167	0.167	0.333	0.167	0.167	
Species		Atom Fraction	Coherent Scattering Length (fm)	2.847	5.803	5.803	9.577	-3.74	
HSO_3Cl	S	0.167	2.847	0.007	0.015	0.029	0.024	0.009	8.5%
	O^{hydr}	0.167	5.803	0.015	0.030	0.060	0.049	0.019	17.3%
	O^{oxo}	0.333	5.803	0.029	0.060	0.120	0.099	0.039	34.6%
	Cl	0.167	9.577	0.024	0.049	0.099	0.081	0.032	28.5%
	H	0.167	-3.74	0.009	0.019	0.039	0.032	0.012	11.1%

Table S7. Fractional neutron scattering contributions of individual atom-atom pairs in the 50:50 HSO_3Cl/DSO_3Cl.

			Species	50:50 HSO_3Cl/DSO_3Cl						Percentage Scattering Contribution
				S	O^{hydr}	O^{oxo}	Cl	H	D	
			Atom Fraction	0.167	0.167	0.333	0.167	0.083	0.083	
Species		Atom Fraction	Coherent Scattering Length (fm)	2.847	5.803	5.803	9.577	-3.74	6.671	
50:50 HSO_3Cl/ DSO_3Cl	S	0.167	2.847	0.007	0.013	0.027	0.022	0.004	0.008	8.1%
	O^{hydr}	0.167	5.803	0.013	0.027	0.055	0.045	0.009	0.016	16.6%
	O^{oxo}	0.333	5.803	0.027	0.055	0.110	0.091	0.018	0.032	33.1%
	Cl	0.167	9.577	0.022	0.045	0.091	0.075	0.015	0.026	27.3%
	H	0.083	-3.74	0.004	0.009	0.018	0.015	0.003	0.005	5.3%
	D	0.083	6.671	0.008	0.016	0.032	0.026	0.005	0.009	9.5%

Table S8. Fractional neutron scattering contributions of individual atom-atom pairs in DSO_3Cl.

			Species	DSO_3Cl S	DSO_3Cl O^{hydr}	DSO_3Cl O^{oxo}	DSO_3Cl Cl	DSO_3Cl D	Percentage Scattering Contribution
			Atom Fraction	0.167	0.167	0.333	0.167	0.167	
Species		Atom Fraction	Coherent Scattering Length (fm)	2.847	5.803	5.803	9.577	6.67	
DSO_3Cl	S	0.167	2.847	0.006	0.012	0.025	0.020	0.014	7.8%
	O^{hydr}	0.167	5.803	0.012	0.025	0.051	0.042	0.029	15.9%
	O^{oxo}	0.333	5.803	0.025	0.051	0.101	0.083	0.058	31.8%
	Cl	0.167	9.577	0.020	0.042	0.083	0.069	0.048	26.2%
	D	0.167	6.67	0.014	0.029	0.058	0.048	0.033	18.3%

Table S9. Fractional X-ray scattering contributions (at 76.6 keV) of individual atom-atom pairs in HSO_3Cl.[8,20]

			Species	X-ray S	X-ray O^{hydr}	X-ray O^{oxo}	X-ray Cl	X-ray H	Percentage Scattering Contribution
			Atom Fraction	0.167	0.167	0.333	0.167	0.167	
Species		Atom Fraction	X-ray form factor	16	8	8	17	1	
X-ray	S	0.167	16	0.074	0.037	0.074	0.079	0.005	26.9%
	O^{hydr}	0.167	8	0.037	0.019	0.037	0.039	0.002	13.4%
	O^{oxo}	0.333	8	0.074	0.037	0.074	0.079	0.005	26.9%
	Cl	0.167	17	0.079	0.039	0.079	0.084	0.005	28.6%
	H	0.167	1	0.005	0.002	0.005	0.005	0.000	1.7%

S.2 Experimental procedure

Materials

Non-deuterated chlorosulfonic acid (99%) and d2-sulfuric acid (96-98 wt. % in D_2O, 99.5 atom % D) were purchased from Sigma Aldrich (UK). Potassium hydroxide (99.9%) was purchased from VWR (UK). Phosphorus pentachloride was purified from a historic sample via sublimation immediately before use. Quartz tubing (6mm ID, 8mm OD) was purchased from Robson Scientific (UK).

Synthesis of chlorosulfonic acid-D_1

Warning: The reagents in this synthesis are highly corrosive. The product, chlorosulfonic acid, is a powerful lachrymator. The reaction is highly exothermic and produces hydrochloric acid and phosphoryl chloride as stoichiometric byproducts. Care must be taken to safely neutralise the byproducts and avoid overpressure of the reaction vessel. Strict air and moisture sensitive techniques were used at all times.

$$DSO_3(OD) + PCl_5 \longrightarrow DSO_3Cl + OPCl_3 + DCl$$

Solid phosphorus pentachloride, PCl_5 (57 g, 277 mmol), was added to a 500 mL 3-necked flask fitted with two taps, a septum and containing a stir bar. The flask was evacuated and placed under nitrogen and an outlet tube connected to a funnel above a saturated solution of KOH (1 L), maintaining a gentle positive flow of nitrogen to prevent backflow into the vessel. To this flask, neat sulfuric acid-D_2, D_2SO_4 (15 mL, 275 mmol), was added dropwise via syringe over the course of 2 hours. Upon starting the addition of D_2SO_4, dinitrogen flow was ceased and resumed once the addition was complete. The reaction was stirred for 2 hours. Phosphoryl chloride, $OPCl_3$, was separated by cold-trap vacuum distillation (30 °C, 0.1 mbar, 4 hours), and the product was purified by further distillation (65 °C, 0.1 mbar). The resultant chlorosulfonic acid-D_1 (*ca*. 10-12 mL) was exposed to dynamic vacuum for 1 hour. The contaminant phosphoryl chloride was quantified by ^{31}P NMR spectroscopy (202 MHz, $CDCl_3$, δ 8.63 ppm) using PPh_3 as an external standard and found to be < 1%. ^{2}H NMR (77 MHz, protic $CHCl_3$): δ 10.63 ppm (<u>D</u>SO_3Cl).

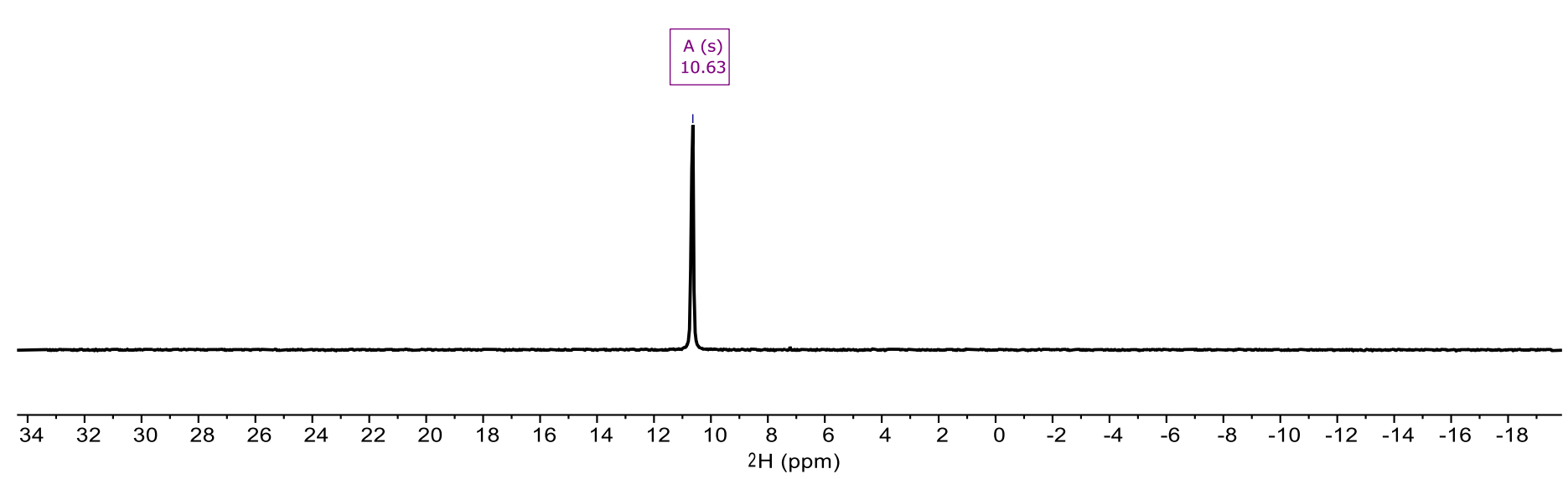

Figure S2. ^{2}H NMR spectrum of the synthetised DSO_3Cl showing the acidic ^{2}D and no contribution from contaminants.

Scattering Measurements

Samples for neutron scattering were prepared by first sealing one end of a quartz tube (6mm ID, 8mm OD, quartz purchased from Robson Scientific) to give a ~15 cm test tube, into which 1.5 mL of chlorosulfonic acid (or 0.75mL of each h-CSA and d-CSA) were added. The samples were sealed with a custom Schlenk-line adapter with Young's tap, quenched in liquid nitrogen, and placed under vacuum before sealing with an oxygen/methane torch to give ~10cm long ampules.

Neutron scattering measurements were performed by mounting ampules in thin foil (0.1mm thick) vanadium cells in the NIMROD diffractometer at ISIS Neutron and Muon Source (UK). Data were collected for a minimum of 160 μA (~4 hours) per sample. Background subtraction was obtained using empty quartz held in vanadium foil, and the data was calibrated against a 8mm diameter vanadium rod using the GudrunN routines. The data processing Q-range was 0.06-50 $Å^{-1}$.

X-ray scattering samples were prepared by injecting (protiated) CSA into a quartz capillary (2mm outer diameter, 0.22mm wall), a standard sample container for the I15-1 beamline at Diamond Light Source (UK). Samples were sealed with cristaseal wax, further sealed with 2-part epoxy glue.

X-ray scattering measurements were undertaken at the I15-1 beamline, acquiring for 10 min at 76.6 keV. Data subtraction was performed using an empty quartz capillary in air using the GudrunX routines and the Q-range for data processing was 0.65-20 $Å^{-1}$.

S.3 Total structure factors ($F(Q)$) and total radial distribution functions ($G(r)$)

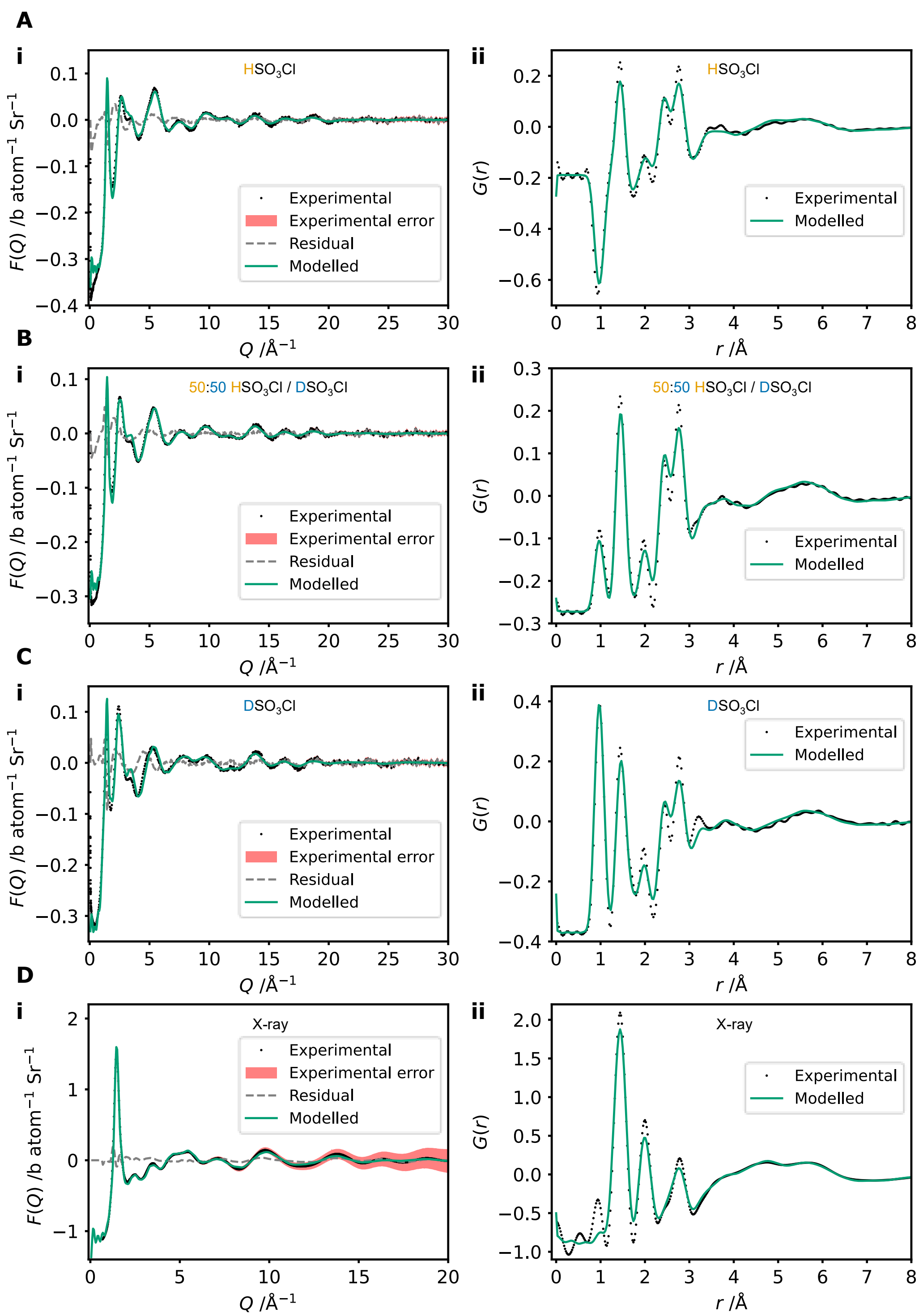

Figure S3. Full F(Q)s and G(r)s of the three isotopic contrasts (A-C) and the X-ray data set (D) with experimental errors.

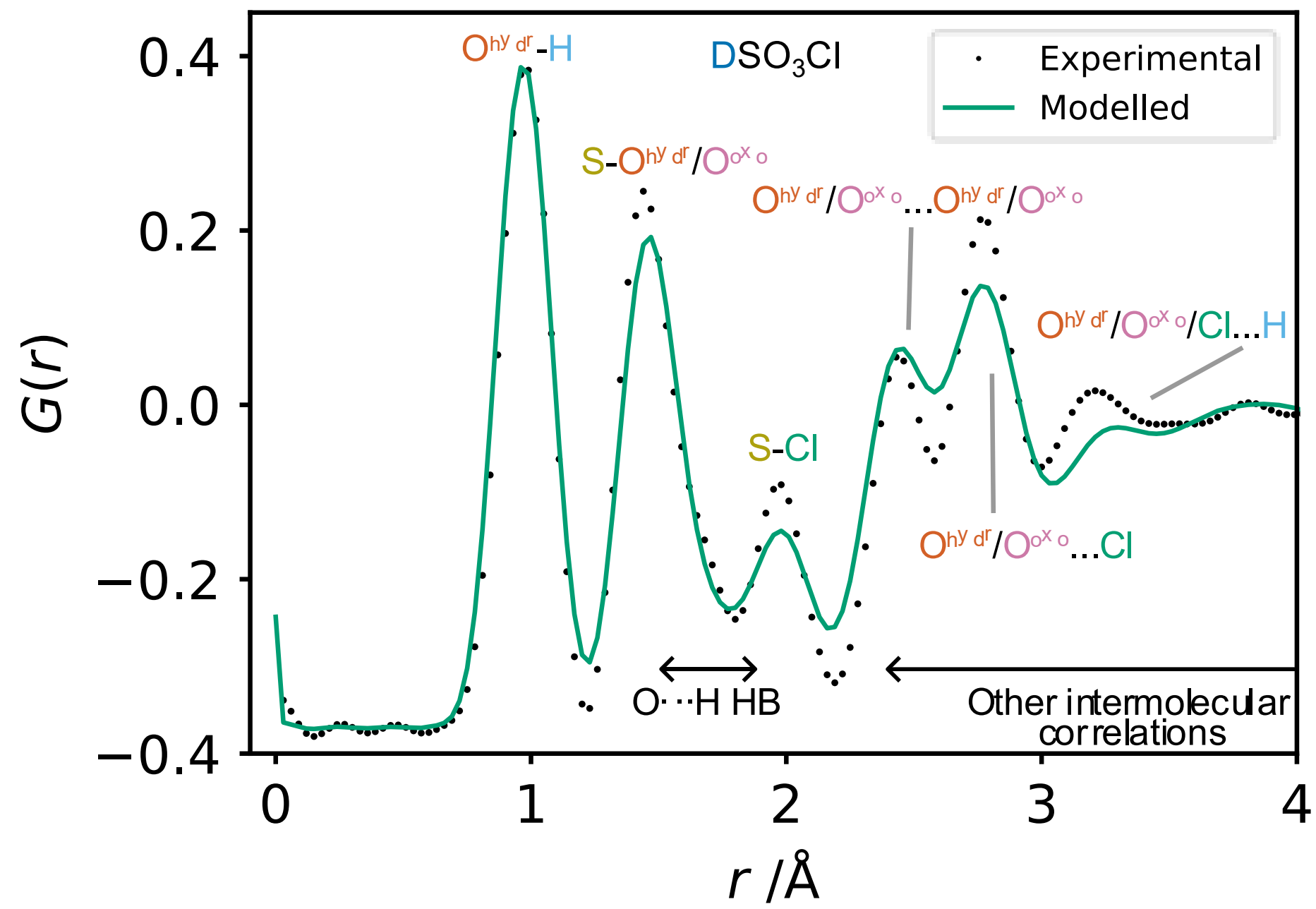

Figure S4. Assigned intramolecular correlations in deuterated CSA. The S-O peak overlaps with contributions from intermolecular hydrogen bonding; other intermolecular correlations are present from ~2.3 Å onwards.

S.4 Radial distribution functions (RDFs) and coordination numbers

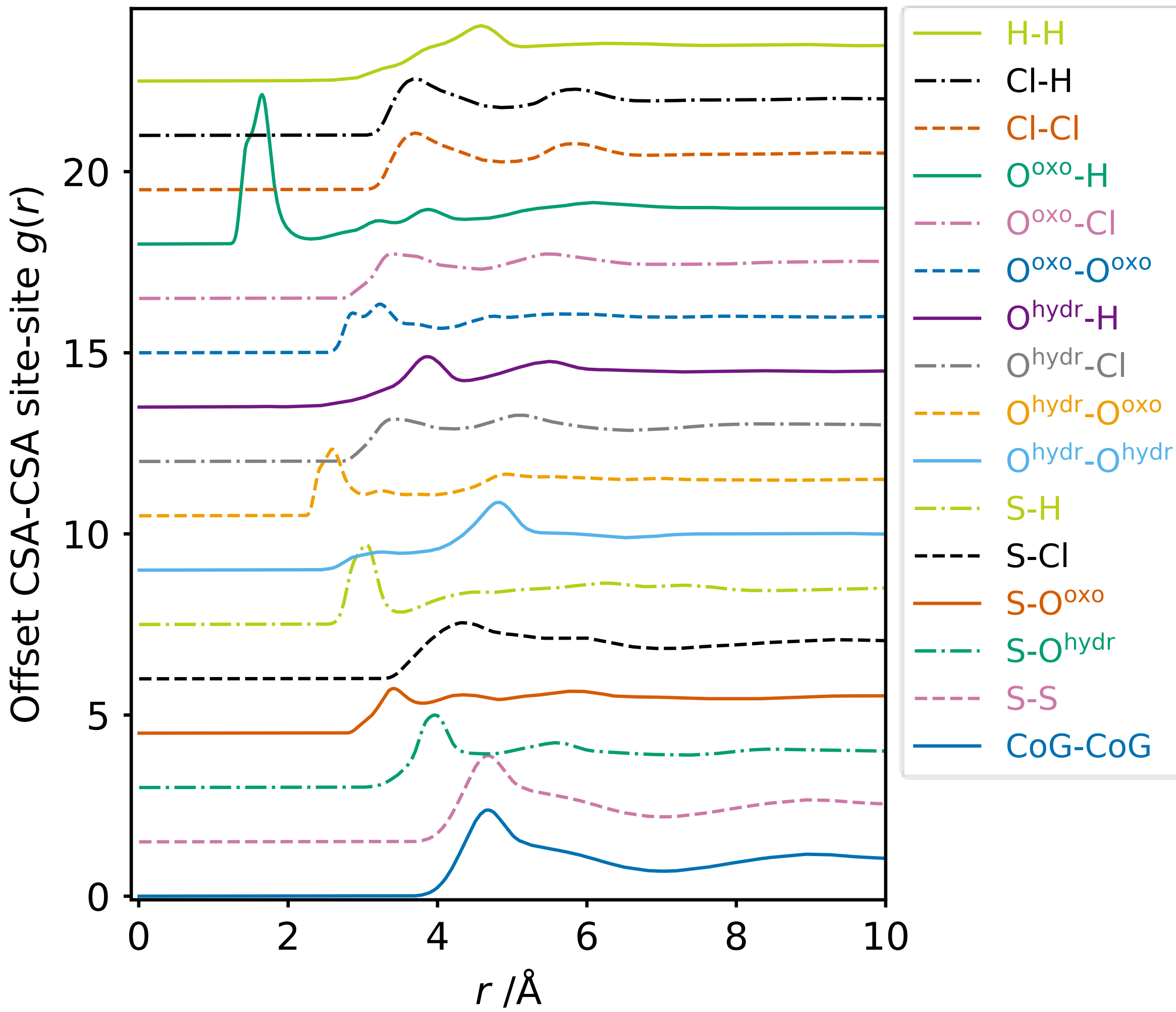

Figure S5. Partial pair distribution functions in chlorosulfonic acid corresponding to all 15 atomic site-site correlations within the system and to the correlation between centres of geometry (CoG).

Table S10. CoG-CoG coordination numbers with cutoffs related to Figure 2b in the Main text and Figure S5 in the SI.

Cutoff /Å	Coordination number
**5.15**	4.35
**7.05**	12.74

S.5 Hydrogen bonding double peak

The double peak corresponding to two separate HB interactions ($O^{oxo}\cdots H$) was seen for fluorosulfonic acid (at slightly further distances, 1.48 Å and 1.74 Å, fitted by two Gaussians), where it was previously assigned to O^{oxo}/F acceptors sites.[21] While the previous work was limited by the poor contrast of F/O, here the use of chlorine and an additional X-ray scattering constraining dataset, as well as significant advances in data analysis[10] indicate that both motifs involve $O\text{-}H\cdots O^{oxo}$ interactions (Main text, Figure 3a-c).

Having two distinct HB interactions to equivalent O^{oxo} is uncommon. However, the feature can be directly confirmed in the experimental data through subtraction of $F_H(Q)$ from $F_D(Q)$ and subsequent Fourier transform to give $\Delta G_{HX}(r)$, which shows only correlations involving hydrogen/deuterium (Figure S6).

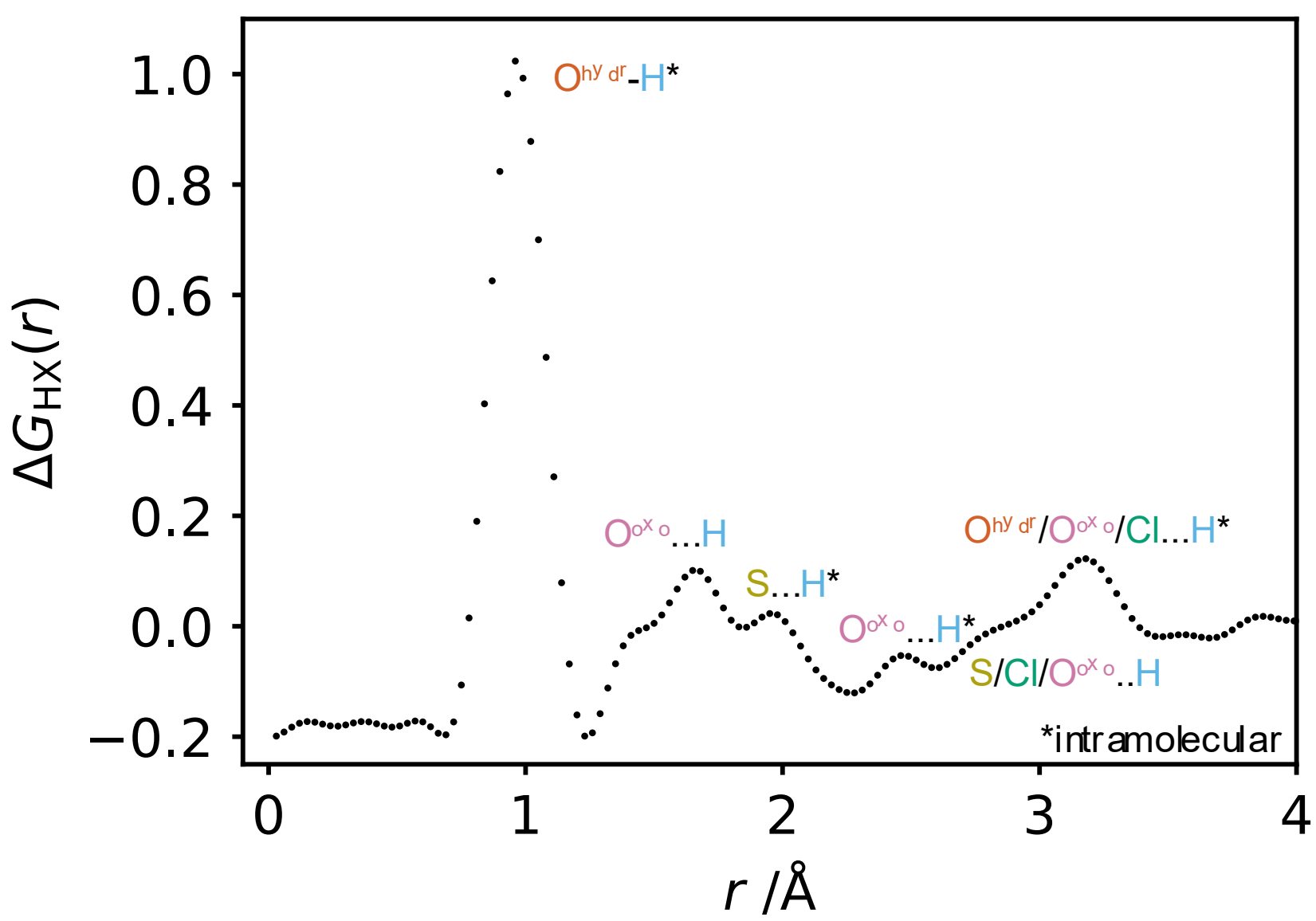

Figure S6. Difference total radial distribution function containing contributions from correlations of H/D atoms. Correlations with an asterisk correspond to intramolecular distances.

By fitting two Gaussian peaks to the $H\cdots O^{oxo}$ partial radial distribution function scaled by $4\pi\rho r^2$, we obtained the peak maxima of 1.43 Å and 1.66 Å, and by taking the *N(r)* up to the the *g(r)* minimum (2.3 Å), we calculated the coordination number of each component giving a 1:5.5 ratio (Figure S7). While Gaussian fitting of $4\pi\rho r^2 g(r)$ is a crude approach which cannot account for several factors including peak asymmetry and a presumption of a known number of environments, the agreement of the fit is very good, even beyond the peak maximum.

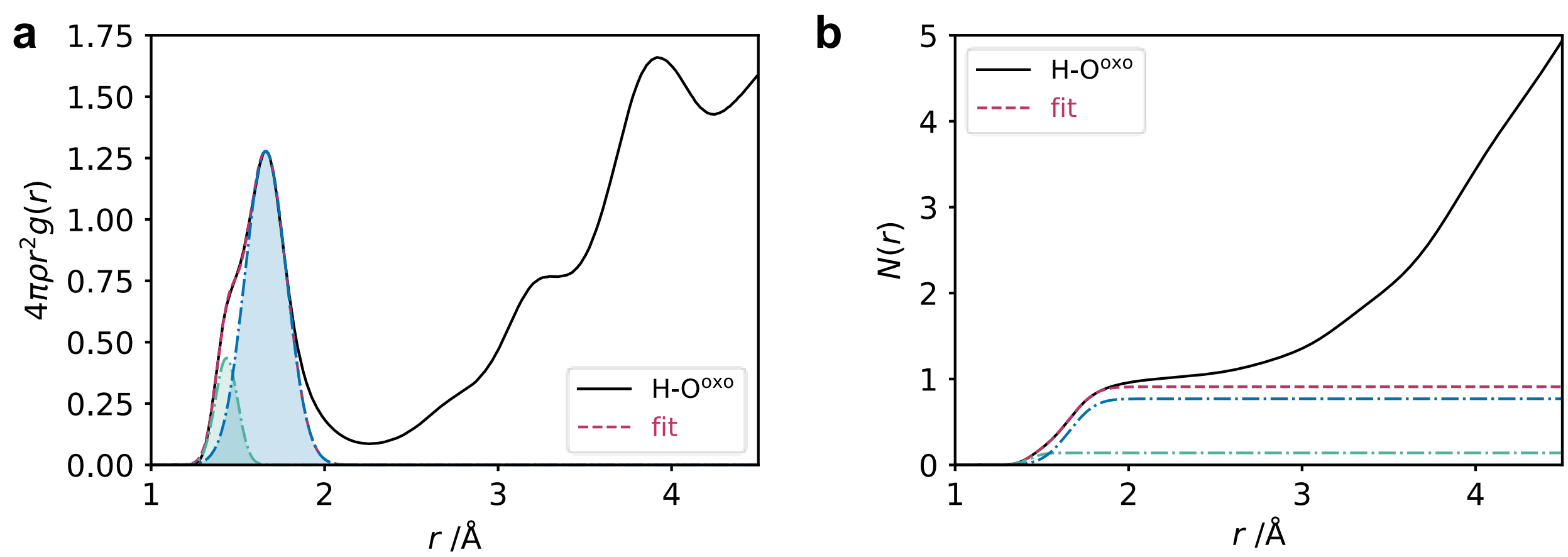

Figure S7. The contributions of the two peaks to O-H···O^{oxo} HB in (a) partial $4\pi\rho r^2 g(r)$, and (b) running coordination number N(r) of O^{oxo} around H. The peak maxima are at 1.43 Å and 1.66 Å, respectively, and the ratio of their areas which corresponds to approximate coordination number ratio is 1:5.5.

The two HB features also exhibit distinct geometric behaviours. In the O-H···O^{oxo} ARDF, the peak at shorter distance is visibly narrower in the angular range as would be expected for a stronger (and hence shorter) interaction. (Figure S8)

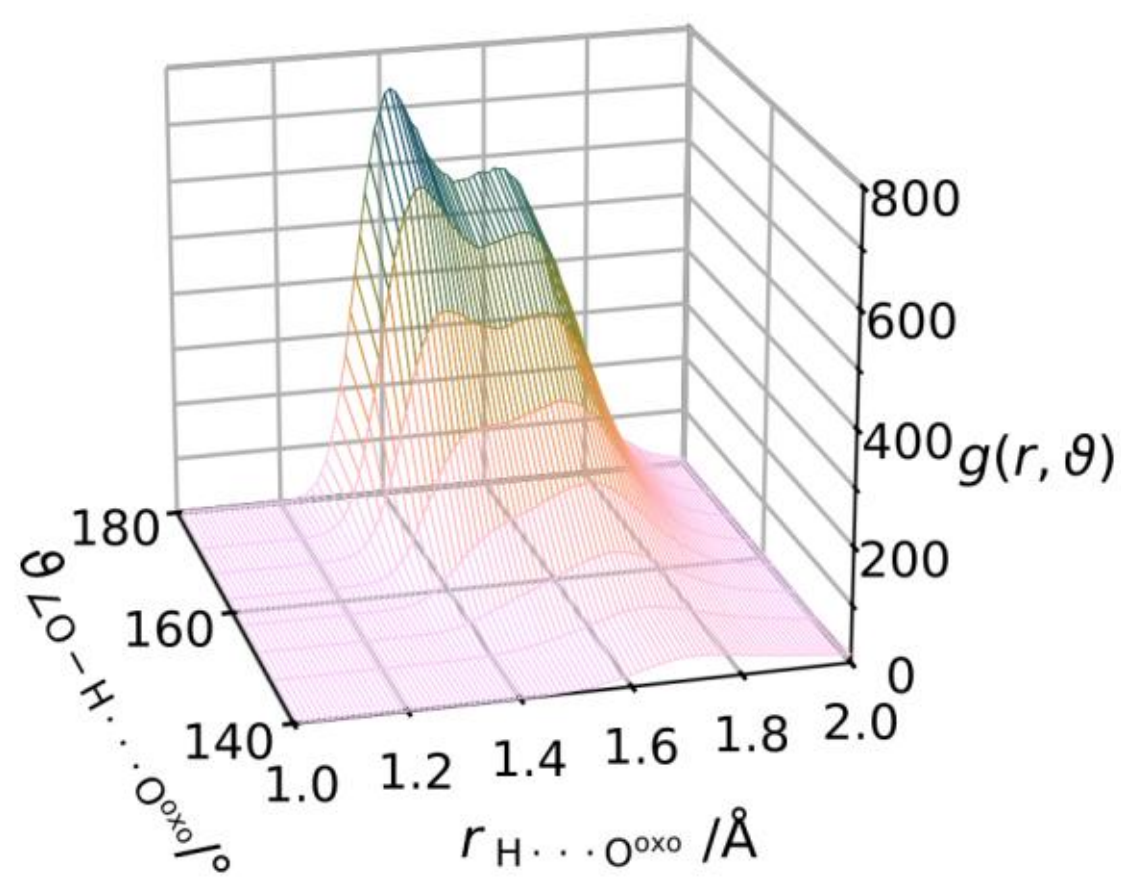

Figure S8. Magnified ARDF of the O-H···O^{oxo} HB double peak showing the sharper nature of the HB peak at shorter distances.

To isolate and confirm the origin of these two features, we have been able rule out causes such as different molecular orientation and effect from the torsional angles. We selected radial cut-offs which maximise differences between the two contributions to give SDF plots of H and O^{hydr} around O^{oxo}, which confirmed there is no orientational bias in either of the O-H···O^{oxo} interactions. (Figure S9). Furthermore, ARDFs of H-O^{oxo}···H and O^{oxo}-H···O^{oxo} feature double peaks regardless of angle, in support of the double peaks not being a consequence of intramolecular torsion. (Figure S10)

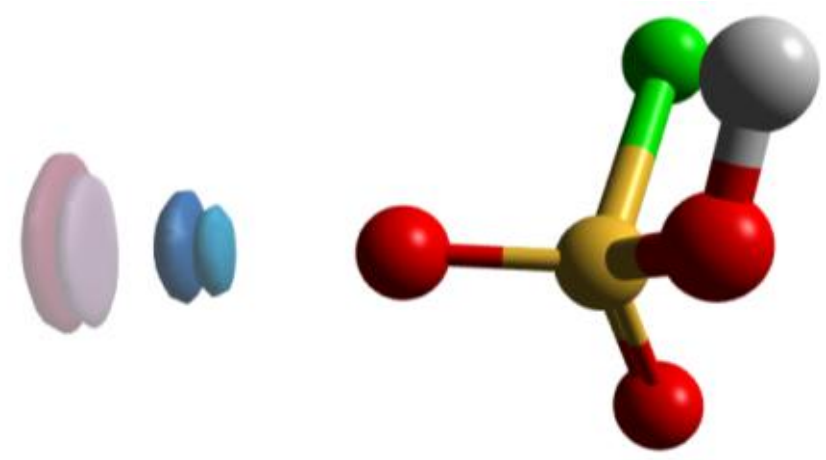

Figure S9. Top 10 % of H (blue) and O^{hydr} (pink) around O^{oxo} corresponding to HB with H···O^{oxo} < 1.53 Å and 1.53-2.30 Å, and O^{hydr}···O^{oxo} < 2.5 Å and 2.5-2.8 Å. The lobes at larger distances are more diffuse but their symmetry does not differ from the closer lobes.

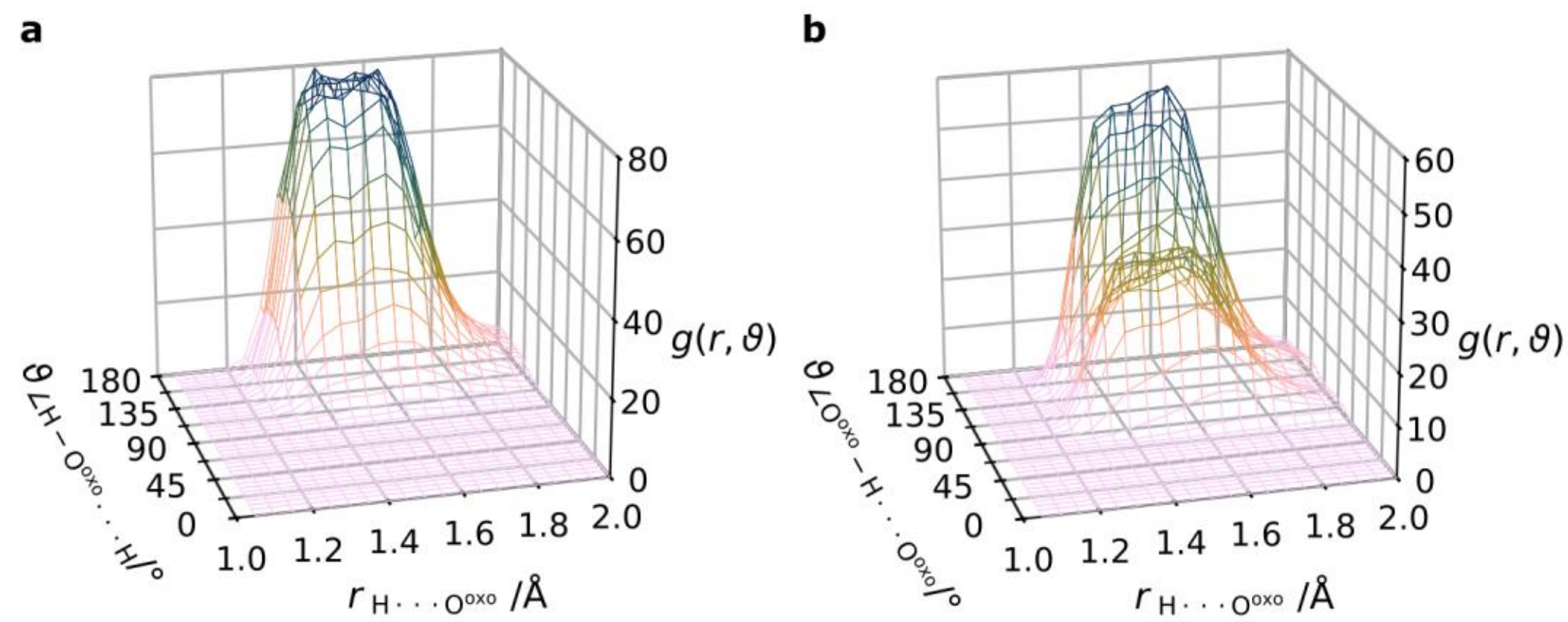

Figure S10. ARDFs of H-O^{oxo}···H and O^{oxo}-H···O^{oxo} correlations showing no angular difference of the two peaks at different distances.

Given the branched chain liquid structure of CSA, we considered a link between the two HB bond distances and the position of a given CSA molecule within the chain. By designating CSA molecules by the number of HBs they were accepting/donating (defined by O^{oxo}···H < 2.0 Å), they could be categorised as being in the linear region of a chain (accepting 1 HB, which is also donating a HB), at a H-terminated chain end (accepting 1 HB, not donating H), at a O-terminated chain end (donating 1 HB, not accepting H), a dimer (only two molecules interacting), or at a branching point (accepting 2 HBs). While the HB interactions at branching points were on average ~0.03-0.04 Å longer than HB within and at the end of linear chains, and ~0.09 Å longer than HB in dimers within the system, this difference is not sufficient to explain the large difference in the two contributions of the HB double peak (0.22 Å). The split nature of the peak is also still mostly preserved in HB length histograms. (Figure S11).

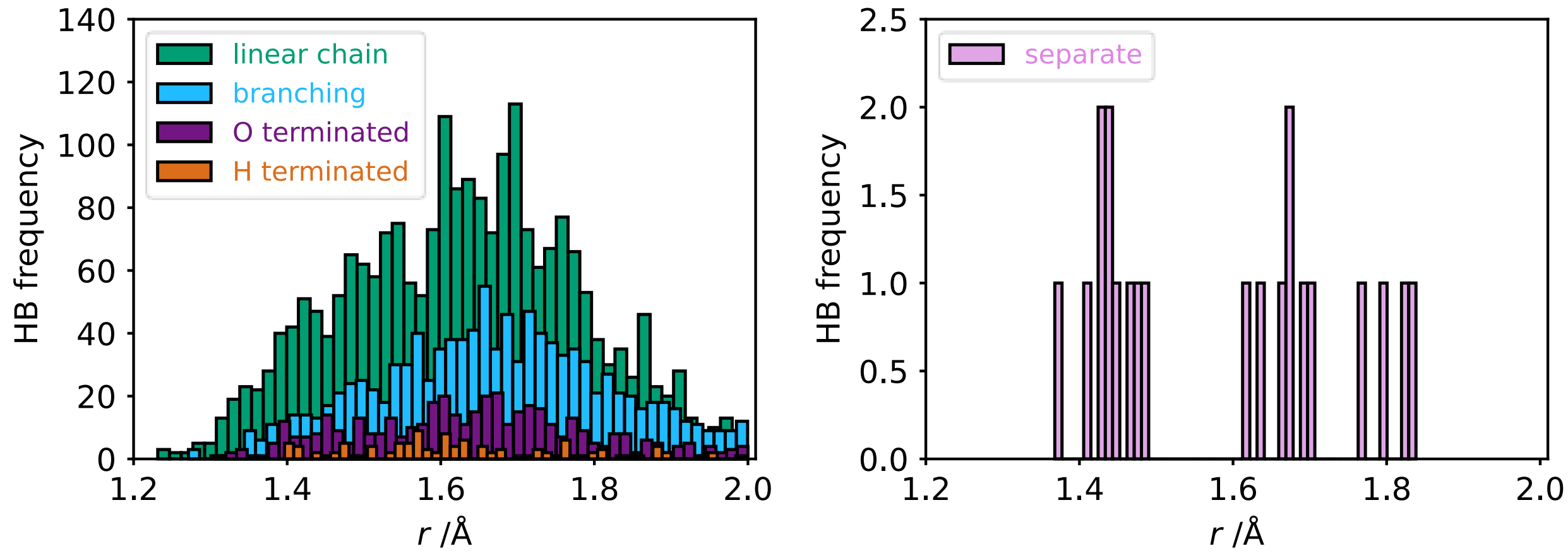

Figure S11. Histograms of HB bond length in linear chains (green), chain branching (blue), at the end of chains (purple/orange), and in CSA dimers from a single snapshot of the simulation box.

Table S11. Average distances of HB interactions based on their position within the molecular chains from a single snapshot of the simulation box. The connectivity was determined with 2.0 Å cutoff for considering HB.

HB position	Average distance /Å
Linear chain	1.63
Branching	1.67
O terminated chain end	1.64
H terminated chain end	1.63
Dimer	1.58

Interestingly, the HB peak ratio changed in liquid fluorosulfonic acid with temperature with the shorter HB more prominent at lower temperatures (ratio 1:2.6 at 193 K compared to 1:4 at 300 K with maxima at 1.48 Å and ~1.73 Å).[21] In solid fluorosulfonic acid measured by X-rays at 123 K, the HB length is ~1.675 Å, very close to the average of the two HB lengths weighted by the peak ratio in liquid state suggesting that either the two distances merge and average out within the solid or the peak is split even in the solid phase. As X-rays are much more sensitive to heavier atoms than H and the double peak is significantly less pronounced in $O^{hydr}\cdots O^{oxo}$, the double peak feature of the HB in the solid state could have been overlooked.[22]

S.6 Angular radial distribution functions (ARDFs)

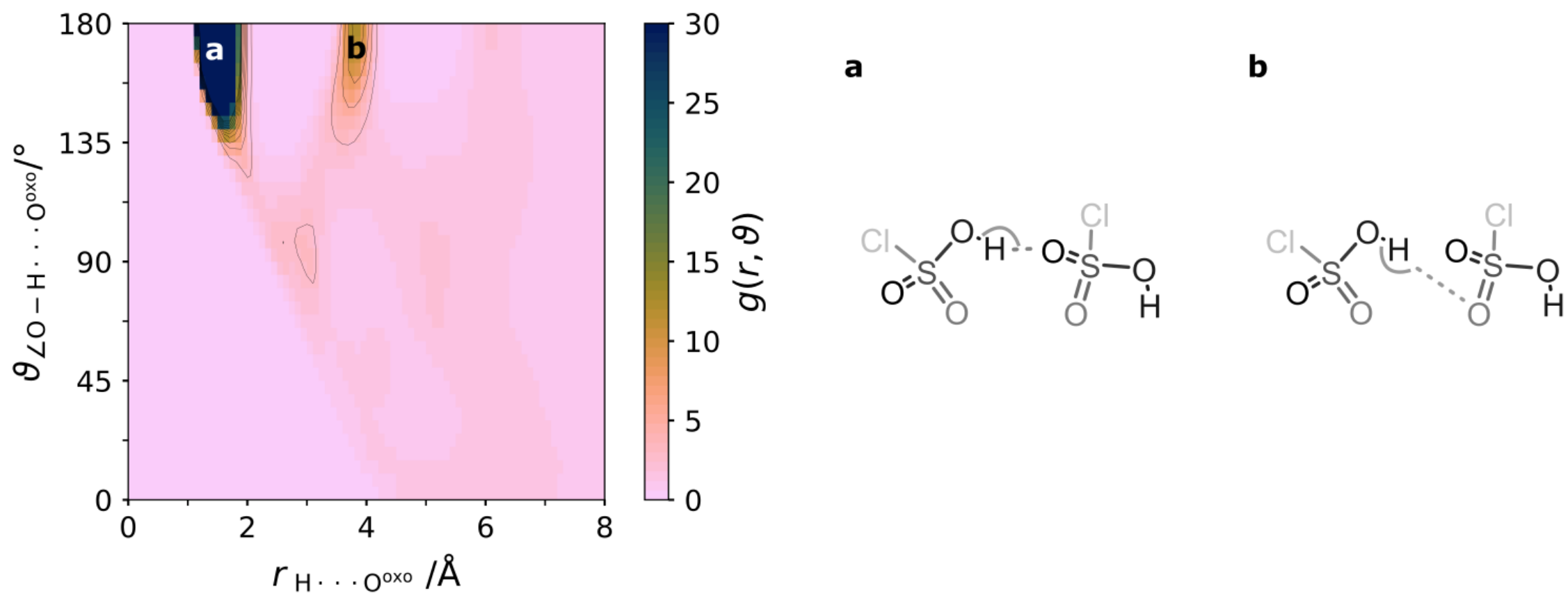

Figure S12. Assignment of peaks (b) in the angle-distance map (a) for H···O^oxo distances and ∠O-H···O^oxo angles. Note the maximum intensity of the angle-distance map was set to 30 to show the correlations other than direct HB, otherwise the maximum is at ~800, see main text Figure 3aii. Hence peak a is saturated and its intensity should not be compared to the intensities of other features.

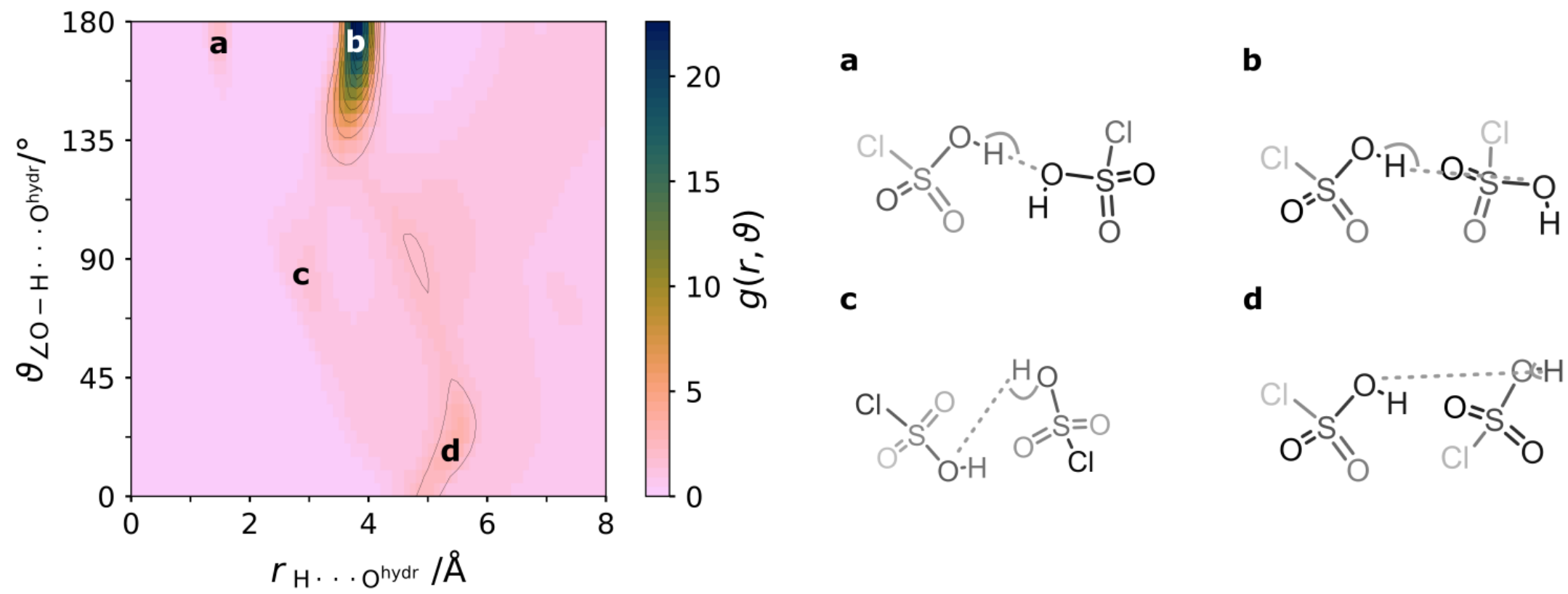

Figure S13. Assignment of peaks (b) in the angle-distance map (a) for H···O^hydr distances and ∠O-H···O^hydr angles. The HB peak is very weak compared to other features. All features can be explained as secondary correlations caused by the presence of strong H···O^oxo HB.

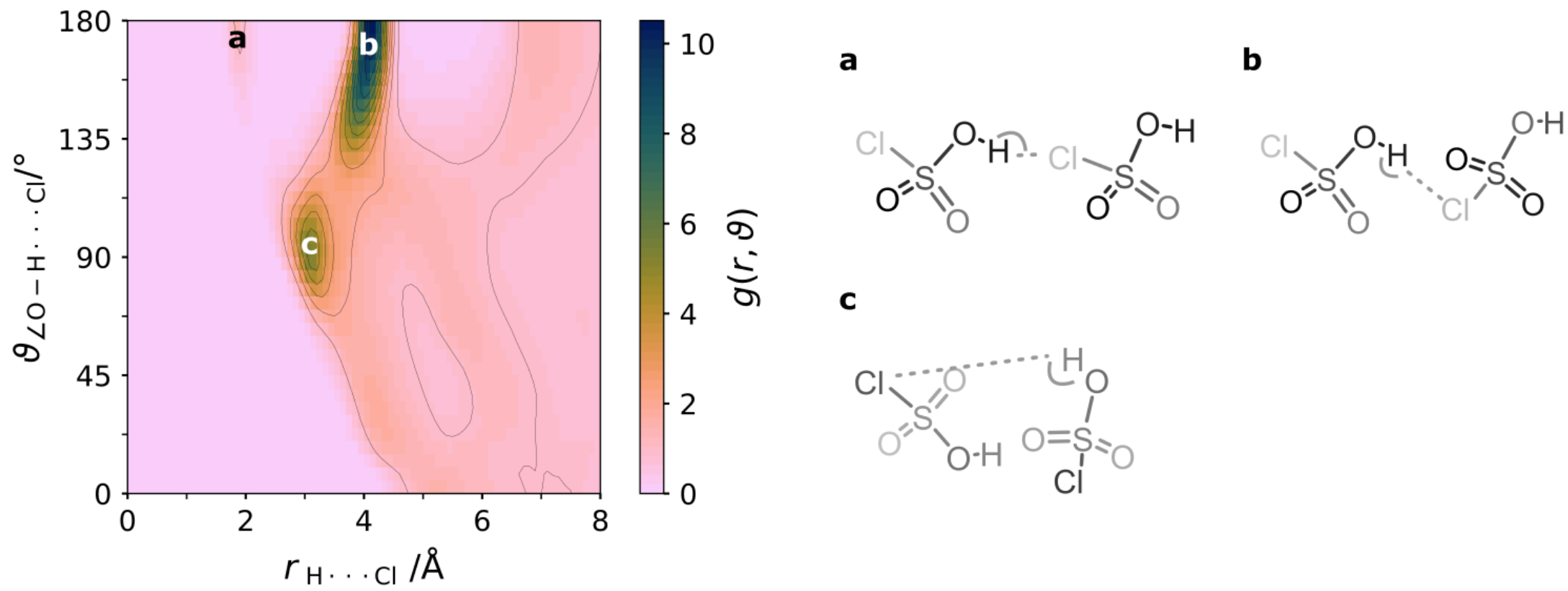

Figure S14. Assignment of peaks (b) in the angle-distance map (a) for H···Cl distances and ∠O-H···Cl angles. The HB peak is very weak compared to other features. All features can be explained as secondary correlations caused by the presence of strong H···O^{oxo} HB.

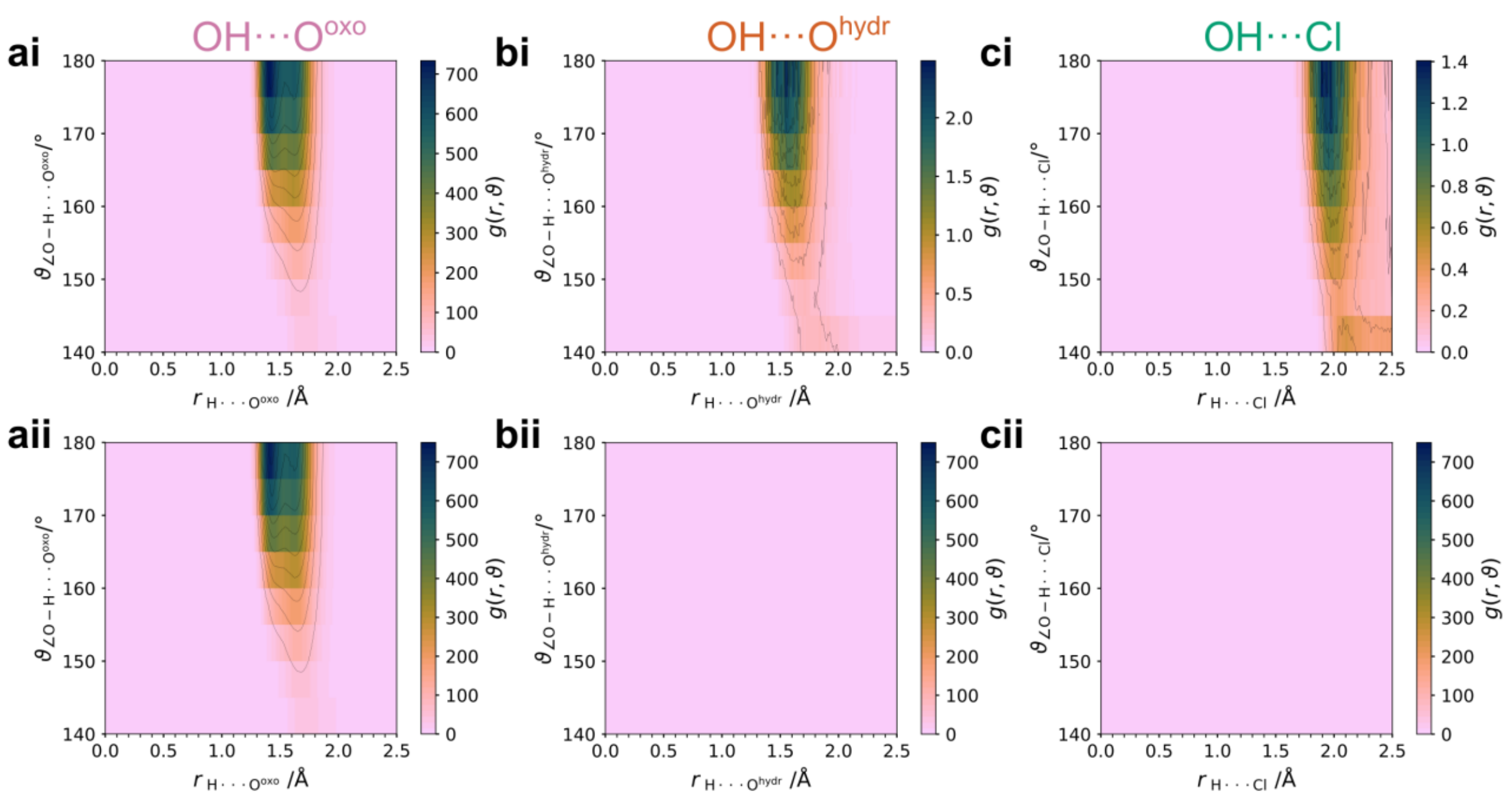

Figure S15. High-angle, short distance HB regions of ARDFs of O-H···O^{oxo} (a), O-H···O^{hydr} (b), and O-H···Cl (c) for O-H···O^{oxo} > 140° and H···O^{oxo} < 2.5 Å. Normalised to maximum peak intensity in ai-ci and plotted on common z-scale in aii-cii.

S.7 Chain formation and HB coordination numbers

CSA chain lengths formed due to HB were generated from the simulation box by setting the maximum $O^{hydr}\cdots O^{oxo}/O^{hydr}/Cl$ distance to 3.35 Å and the minimum O-H··· $O^{oxo}/O^{hydr}/Cl$ angle to 150°. There were 128 clusters in a simulation box snapshot with 3663 molecules with an average cluster size 32.7 CSA molecules (0.89 %), and the largest cluster consisted of 615 molecules (16.8 %) while 25 molecules did not participate in any HB (0.68 %).

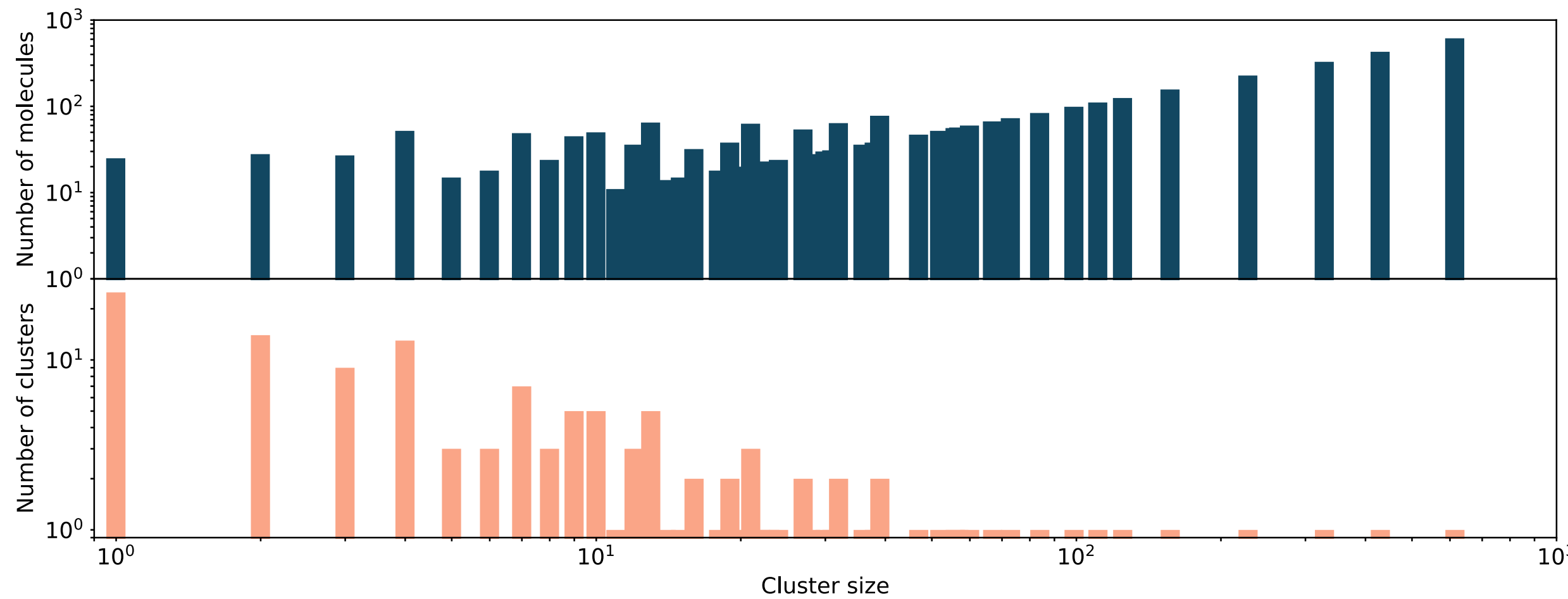

Figure S16. Distribution of cluster sizes of HB CSA molecules within simulation box snapshot.

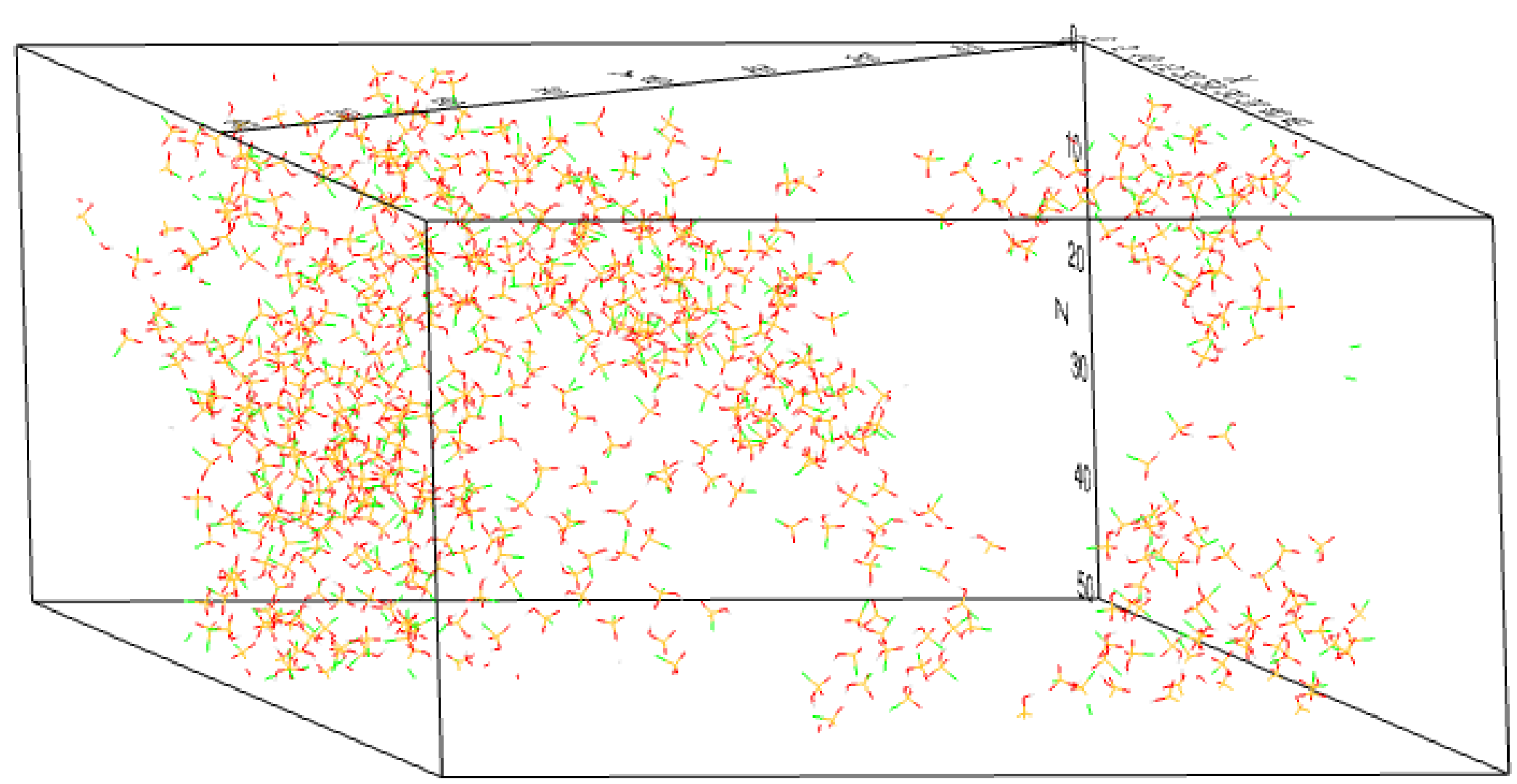

Figure S17. 3D snapshot of a chain formed by HB between 491 CSA molecules in a simulation box with 3663 molecules. This cluster has been the largest found in the simulation box.

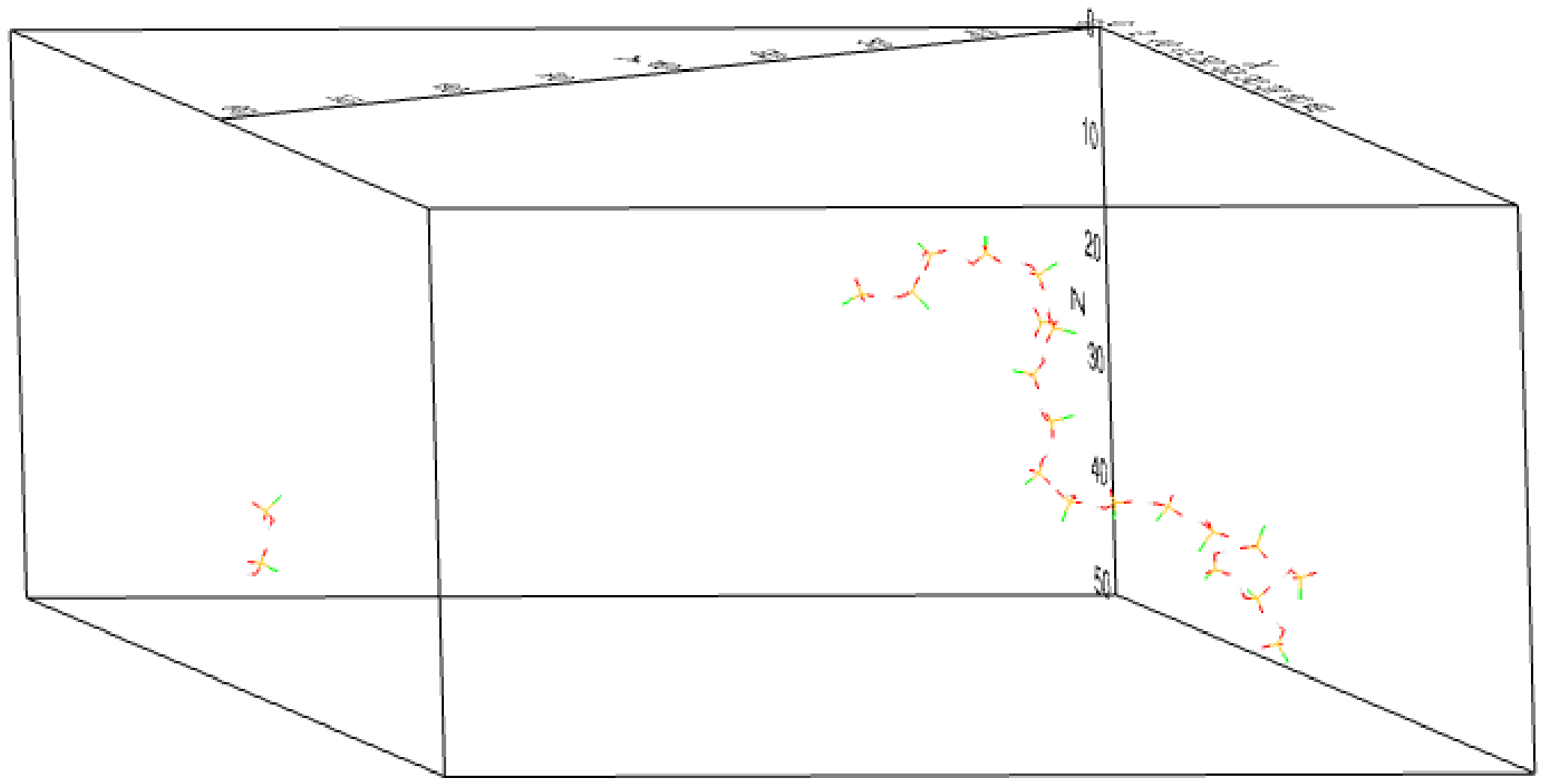

Figure S18. 3D snapshot of a chain formed by HB between 24 CSA molecules in a simulation box with 3663 molecules. This cluster shows very well the branched chains consisting of the CSA molecules.

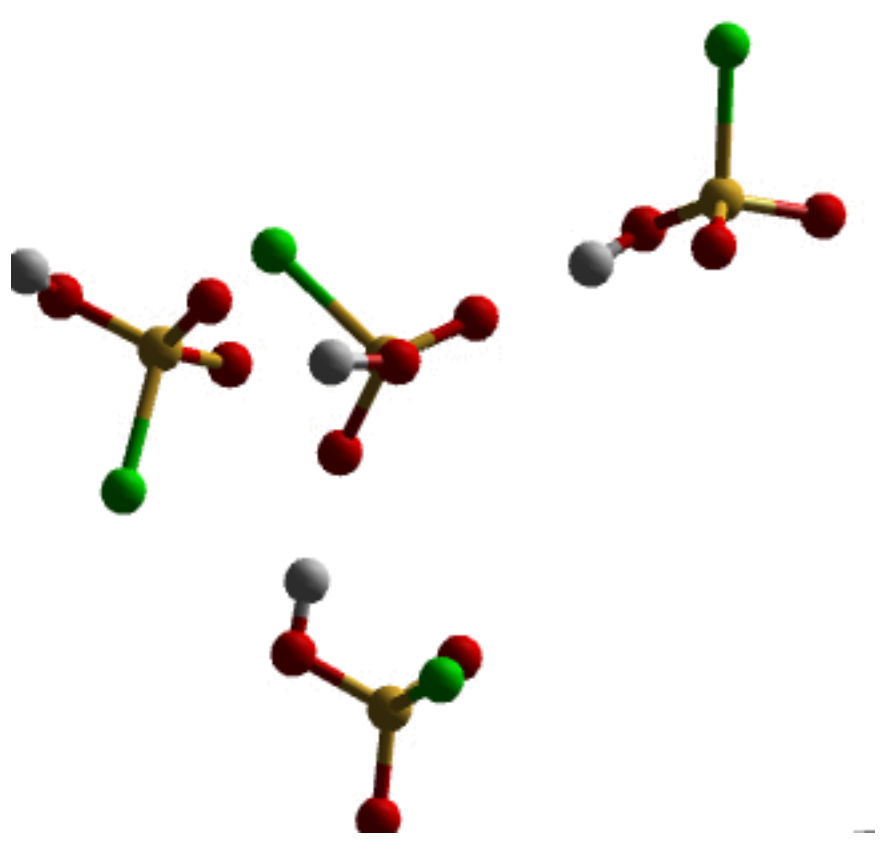

Figure S19. CSA molecules forming a branch in the chain of molecules. The molecule where the branching occurs forms three HB to surrounding molecules – one via H (HB donor) and two via O^{oxo} (HB acceptors).

Table S12. Average number of S atoms with 0, 1, 2, and 3 H atoms closer than 3.50 Å and the related coordination number.

Number of H	Average number of contacts per site
**0**	0.153
**1**	0.658
**2**	0.188
**3**	0.001
**Average CN**	1.037

References

(1) Soper, A. *GudrunN and GudrunX: Programs for Correcting Raw Neutron and X-Ray Diffraction Data to Differential Scattering Cross Section*; Science & Technology Facilities Council, 2012.

(2) Sivia, D. S. The Basics of X-Ray and Neutron Scattering. In *Elementary Scattering Theory: For X-ray and Neutron Users*; Sivia, D. S., Ed.; Oxford University Press, 2011; p 0. https://doi.org/10.1093/acprof:oso/9780199228676.003.0003.

(3) Bowron, D. T.; Soper, A. K.; Jones, K.; Ansell, S.; Birch, S.; Norris, J.; Perrott, L.; Riedel, D.; Rhodes, N. J.; Wakefield, S. R.; Botti, A.; Ricci, M.-A.; Grazzi, F.; Zoppi, M. NIMROD: The Near and InterMediate Range Order Diffractometer of the ISIS Second Target Station. *Rev. Sci. Instrum.* **2010**, *81* (3), 033905. https://doi.org/10.1063/1.3331655.

(4) *ISIS NIMROD*. https://www.isis.stfc.ac.uk/Pages/nimrod.aspx (accessed 2025-11-25).

(5) *XPDF (I15-1) - - Diamond Light Source*. https://www.diamond.ac.uk/Instruments/Crystallography/I15-1.html (accessed 2025-11-26).

(6) Connolley, T.; Beavers, C. M.; Chater, P. High-Energy Adventures at Diamond Light Source. *Synchrotron Radiat. News* **2020**, *33* (6), 31–36. https://doi.org/10.1080/08940886.2020.1841494.

(7) Sears, V. F. Neutron Scattering Lengths and Cross Sections. *Neutron News* **1992**, *3* (3), 26–37. https://doi.org/10.1080/10448639208218770.

(8) Waasmaier, D.; Kirfel, A. New Analytical Scattering-Factor Functions for Free Atoms and Ions. *Acta Crystallogr. Sect. A* **1995**, *51* (3), 416–431. https://doi.org/10.1107/S0108767394013292.

(9) Soper, A. K. Empirical Potential Monte Carlo Simulation of Fluid Structure. *Chem. Phys.* **1996**, *202* (2), 295–306. https://doi.org/10.1016/0301-0104(95)00357-6.

(10) Youngs, T. Dissolve: Next Generation Software for the Interrogation of Total Scattering Data by Empirical Potential Generation. *Mol. Phys.* **2019**, *117* (22), 3464–3477. https://doi.org/10.1080/00268976.2019.1651918.

(11) Chlorosulfonic-Acid-Push-Information.Pdf. https://info.veolianorthamerica.com/hubfs/offers/info-sheets/industrial/chlorosulfonic-acid-push-information.pdf (accessed 2025-11-18).

(12) Jorgensen, W. L.; Tirado-Rives, J. Potential Energy Functions for Atomic-Level Simulations of Water and Organic and Biomolecular Systems. *Proc. Natl. Acad. Sci.* **2005**, *102* (19), 6665–6670. https://doi.org/10.1073/pnas.0408037102.

(13) Dodda, L. S.; Vilseck, J. Z.; Tirado-Rives, J.; Jorgensen, W. L. 1.14*CM1A-LBCC: Localized Bond-Charge Corrected CM1A Charges for Condensed-Phase Simulations. *J. Phys. Chem. B* **2017**, *121* (15), 3864–3870. https://doi.org/10.1021/acs.jpcb.7b00272.

(14) Dodda, L. S.; Cabeza de Vaca, I.; Tirado-Rives, J.; Jorgensen, W. L. LigParGen Web Server: An Automatic OPLS-AA Parameter Generator for Organic Ligands. *Nucleic Acids Res.* **2017**, *45* (W1), W331–W336. https://doi.org/10.1093/nar/gkx312.

(15) Valeev, E. F. *Libint: A library for the evaluation of molecular integrals of many-body operators over Gaussian functions, Version 2*. http://libint.valeyev.net/ (accessed 2025-12-18).

(16) Lehtola, S.; Steigemann, C.; Oliveira, M. J. T.; Marques, M. A. L. Recent Developments in Libxc — A Comprehensive Library of Functionals for Density Functional Theory. *SoftwareX* **2018**, *7*, 1–5. https://doi.org/10.1016/j.softx.2017.11.002.

(17) Weigend, F.; Köhn, A.; Hättig, C. Efficient Use of the Correlation Consistent Basis Sets in Resolution of the Identity MP2 Calculations. *J. Chem. Phys.* **2002**, *116* (8), 3175–3183. https://doi.org/10.1063/1.1445115.

(18) Neese, F. The ORCA Program System. *WIREs Comput. Mol. Sci.* **2012**, *2* (1), 73–78. https://doi.org/10.1002/wcms.81.

(19) Reynolds, A. J.; Rodriguez, D. E.; Lin, W.; Leopold, K. R. Microwave Spectroscopy and Large Amplitude Motion of Chlorosulfonic Acid (ClSO2OH). *J. Mol. Spectrosc.* **2024**, *403*, 111927. https://doi.org/10.1016/j.jms.2024.111927.

(20) Chantler, C. T. Theoretical Form Factor, Attenuation, and Scattering Tabulation for Z=1–92 from E=1–10 eV to E=0.4–1.0 MeV. *J. Phys. Chem. Ref. Data* **1995**, *24* (1), 71–643. https://doi.org/10.1063/1.555974.

(21) McLain, S. E.; Benmore, C. J.; Turner, J. F. C. The Structure of Liquid Fluorosulfuric Acid Investigated by Neutron Diffraction. *J. Chem. Phys.* **2002**, *117* (8), 3816–3821. https://doi.org/10.1063/1.1495395.

(22) Bartmann, K.; Mootz, D. Structures of Two Strong Brønsted Acids: (I) Fluorosulfuric Acid and (II) Trifluoromethanesulfonic Acid. *Acta Crystallogr. C* **1990**, *46* (2), 319–320. https://doi.org/10.1107/S0108270189010395.
